# Adherence to ecological momentary assessment studies in children and adolescents with psychopathology: A systematic review with meta-analysis

**DOI:** 10.1038/s44277-026-00058-z

**Published:** 2026-04-27

**Authors:** Lauren M. Henry, Urmi Pandya, Eleanor Malone, Caroline Miller, Savana Agyemang, Elizabeth Tandilashvili, Kolin Lewis, Clara E. Haeffner, Kyunghun Lee, Alicia A. Livinski, Miryam Kiderman, Reut Naim, Olivia Metcalf, Silvia Lopez-Guzman, Melissa A. Brotman

**Affiliations:** 1 Emotion and Development Branch, National Institute of Mental Health (NIMH), Bethesda, MD USA; 2 National Institutes of Health Library, Office of Research Services, Office of the Director, NIH, Bethesda, MD USA; 3 The School of Psychological Sciences, Tel-Aviv University, Tel-Aviv, Israel; 4 Phoenix Australia, Department of Psychiatry, The University of Melbourne, Melbourne, Australia

**Keywords:** Human behaviour, Human behaviour, Social behaviour

## Abstract

Ecological momentary assessment (EMA) is a tool facilitating the repeated collection of real-time data in naturalistic settings that can be applied to clinical research. Despite established methodological strengths, EMA protocols may be burdensome for participants, potentially leading to problems with adherence. Existing meta-analytic evidence is insufficient to inform the decision making of clinical researchers in using EMA in their work. In the current registered report, we address this need by conducting a systematic review with meta-analysis (1) examining adherence (enrollment, dropout, and compliance) in children and adolescents with psychological disorders and symptoms and (2) identifying aspects of study design that maximize adherence. In January 2023, we searched five databases for empirical EMA studies of youth (8–17 years) with psychological symptoms/diagnoses with data on adherence. We extracted data, assessed study quality, and conducted meta-analyses using random-effects models. We included 130 studies with 14,400 participants. On average, surveys contained 15 items, took over 3 min to complete, and incorporated surveys with multiple response scale types delivered using smartphones/cell phones provided to participants. Meta-analyses revealed that study enrollment was moderate to high (82% in youth with psychopathology, 67% in community youth) and dropout prior to starting EMA protocols was low (<1–2%). Dropout during EMA protocols was 12% for youth with psychopathology and 8% for community youth, underscoring the importance of maintaining participant engagement throughout EMA studies. Compliance approximated the 80% field benchmark for healthy youth but was lower for youth from the community and with psychopathology. Participant characteristics (e.g., younger age) and design factors (e.g., incentive-based compensation) were associated with greater compliance in certain groups. Despite limitations (missing data, methodological constraints), findings have important implications for researchers developing and conducting pediatric EMA protocols. The stage 1 protocol for this Registered Report was accepted in principle on April 19, 2024. The protocol, as accepted by the journal, can be found at: 10.6084/m9.figshare.25732482.v1. This research was supported by the Intramural Research Program (IRP) of the NIMH and the NIH Library’s and Office of Research Service’s support of the NIH IRP.

## Lay summary

Ecological momentary assessment (EMA) is a tool for collecting naturalistic, real-time data. We conducted a systematic review with meta-analysis examining adherence (enrollment, dropout, and compliance) in children and adolescents with psychological disorders and symptoms and aspects of study design that maximize adherence.

## Ecological momentary assessment (EMA) in pediatric clinical research

Published research using ecological momentary assessment (EMA) has increased by 168% over the past decade [1]. EMA is a robust tool for measuring emotions, cognition, and behavior naturalistically through real-time and repeated assessments [2]. While EMA has unique advantages [3], researchers face challenges with participant protocol adherence [4]. Such adherence problems may be magnified in youth, particularly in children struggling with symptoms of psychopathology [5]. While specific study design choices may optimize adherence [6], existing meta-analytic evidence is insufficient to inform decision making in youth with psychopathology. In a systematic review with meta-analysis, we provide an overview of existing EMA protocols for youth with psychopathology, and we address this research gap by (1) examining adherence (enrollment, dropout, and compliance) in children and adolescents with a wide range of psychological disorders and symptoms and (2) identifying aspects of study design that maximize adherence.

## Advantages and challenges of EMA research

EMA designs are advantageous for pediatric clinical research. EMA allows investigators to collect data with high levels of ecological validity (relative to questionnaire and lab-based methods [3]) and examine dynamic, within-person processes across time [7]. EMA may be particularly useful for clinical conditions with components that evolve quickly and are thus difficult to assess and monitor using traditional methods (e.g., suicidal ideation [8]). Further, EMA provides an increasingly seamless pathway toward translation of basic research findings to clinical intervention delivered at scale through ecological momentary interventions or just-in-time adaptive interventions [9].

At the same time, EMA has limitations [2, 10]. EMA may be prone to recall biases depending on item coverage (e.g., “since the last beep”), though the impact is lessened relative to questionnaires with reporting windows across weeks and months [2, 10]. Further, EMA may be subject to measurement reactivity (change in behavior as a consequence of assessment) [11, 12], and EMA cannot be used to make claims about causality in the context of non-experimental research designs [13]. Issues associated with technology and its use (e.g., software bugs, uncharged devices) have implications for missing data and should be considered by researchers [14].

The established benefits of EMA research may be further diminished due to low adherence [15, 16]. Participant perceptions or experiences of burden may impact adherence metrics, including consenting to participate in a study (enrollment), persisting in a study once enrolled (dropout), or completing administered surveys (compliance) [17]. To date, no meta-analytic research on adherence in pediatric samples has reported enrollment (only dropout and compliance [18]); examined adherence across psychological disorders and symptoms (only broadly-defined “clinical samples” or discrete diagnostic groups [19]); or tested aspects of study design as moderators of adherence across psychological disorders and symptoms.

## Enrollment, dropout, and compliance in EMA studies

Three metrics can assess participant adherence to an EMA study: enrollment, dropout, and compliance. Enrollment (also referred to as response [20]) is the proportion of recruited participants who consent to enroll in a study. Dropout (also referred to as attrition or retention [6, 20]) is the proportion of enrolled participants who exhibit nonresponse prior to study completion. Compliance (also referred to as completion [21]) is the proportion of prescribed surveys that are responded to by participants.

**Enrollment** is an adherence metric infrequently reported in EMA studies. In the only EMA meta-analysis reporting enrollment, Morren et al. [20] found that just over half (53%) of individuals recruited into EMA studies (here, primarily adults) decided to enroll. However, only 12 of 43 studies (28%) included in the meta-analysis reported study enrollment [20]. As such, infrequent reporting of enrollment in meta-analytic research may be related to infrequent reporting of enrollment in EMA studies.

**Dropout** is an adherence metric more frequently (though not commonly) reported, both in meta-analytic research and individual EMA studies. Meta-analyses have found participant dropout to range from 7% [6] to 19% [20], with meta-analyses of youth and adult samples finding no differences based on age [4, 22]. The number of individual EMA studies reporting dropout ranges from 28% (140 of 496 individual included studies [22]) to 70% (30 of 43 individual included studies [20]).

**Survey compliance** is the most commonly reported adherence metric in both EMA meta-analyses and individual EMA studies. Eighty percent is an agreed-upon (though not necessarily empirically justified) benchmark for “adequate” survey compliance [19]. Most meta-analytic research in youth and adults reports compliance close to 80% [4, 22]. However, importantly, there is variation in compliance across EMA studies [4, 21].

## Factors associated with adherence in EMA studies

Previous meta-analyses have probed the heterogeneity in EMA compliance [22], and aspects of study design have been examined as moderators [23]. Individual differences among participants, particularly in psychological symptoms and disorders, may also be related to adherence [6].

### Study design

There are multiple aspects of study design to be considered in EMA research [1]. Investigators make choices regarding their sampling scheme (e.g., fixed, semirandom, random), scheduling (e.g., number of assessment days, number of surveys per day, survey time of day, time between surveys), survey features (e.g., scale types, number of items, duration, use of alarms), type of platform and delivery device, provision of individualized feedback on compliance, compensation (e.g., amount, type), and level of contact with researchers during the study. While motivations for design decisions vary (e.g., guidance from previous research and experts, nuances of individual research questions, feasibility [23]) and may be more or less adjustable, these choices may have consequences for adherence.

In review, “less burdensome” protocols are related to greater compliance, including fewer total assessment days [4, 21], fewer surveys per day [4, 6], shorter surveys [20], and longer time intervals between successive surveys [6]. The larger context of the study (i.e., concurrent participant burdens) should also be considered, with evidence of lower compliance and higher dropout in intervention studies [4]. Design choices that make participation more transparent and rewarding are associated with greater compliance. Cueing participant awareness to surveys using alarms and fostering participant understanding of study protocols through the provision of manuals is supported in meta-analytic research [20]. Sampling scheme choices may also be relevant, as Vachon et al. [6] demonstrated higher compliance in studies with fixed relative to random and semirandom sampling. Modes of survey delivery may also be considered, with Vachon et al. [6] finding the use of Web-based and mixed data collection methods to be associated with greater compliance as compared to personal digital assistants (PDAs). Finally, incentives have been shown to be relevant to both compliance and dropout (e.g., higher value monetary incentives have been associated with greater compliance) [6, 20, 22].

### Psychological symptoms and disorders

In comparing clinical to nonclinical populations broadly, meta-analyses have found few and small differences in EMA compliance across groups [4, 22, 24]. However, psychopathology is vastly heterogeneous [25], and meta-analyses that have parsed psychopathology by diagnosis have found differences in compliance across disorders. For example, compliance has been shown to be reduced in adults with psychosis relative to individuals with other disorders and no disorders [5, 6] and adolescents and adults with substance use disorder relative to those without diagnoses [19]. Accordingly, additional meta-analytic research is needed to understand variation in adherence by diagnostic type to inform clinical research.

## Study designs to maximize EMA adherence in youth with psychological symptoms and disorders

Questions remain regarding how EMA study design impacts adherence in youth with psychological symptoms and disorders. With additional information about the intersection of clinical characteristics and study design, clinical researchers may be able to optimize research in their populations of interest by simultaneously maximizing opportunities for data collection while minimizing participant burden. Broadly, Wen et al. [24] found compliance to be higher in clinical populations when surveys were more frequent (6 or more times daily) and higher in nonclinical populations when surveys were less frequent (2–3 times daily). Still, there may be specificity reflecting optimal study designs for certain diagnostic groups. For example, in a pooled sample of 10 studies, those studies pairing EMA with saliva sampling showed greater compliance in individuals with psychosis as compared to studies without saliva sampling [5]. In addition, in a meta-analysis of adolescents and adults with substance use, Jones et al. [19] found that the inclusion of event-contingent assessment (i.e., participant ability to self-initiate a survey at the time that a specific event occurs, such as smoking a cigarette) was associated with greater compliance.

## The current research

### Objectives

In this registered report, our aim was to conduct a systematic review with meta-analysis to provide an overview of existing EMA protocols for youth with psychopathology, determine adherence (enrollment, dropout, and compliance) to EMA studies for children and adolescents (ages 8–17) with psychological disorders and psychological symptoms, and provide insight into which study designs are the most effective (i.e., maximizing enrollment, minimizing dropout, maximizing compliance) for whom (e.g., youth with anxiety, depression). To this end, we answered the following research questions:

1. Primary research question: Among youth with psychological disorders and symptoms, what are the estimates for (1) enrollment, (2) dropout, (3) compliance?
2. Secondary research question: Conditional upon sufficient heterogeneity in effects, which aspects of study design (1) maximize enrollment, (2) minimize dropout, and (3) maximize compliance in youth with psychological disorders and symptoms?

Given that individual differences have been shown to impact enrollment, dropout, and compliance [6, 20–22], we also examined age and sex as moderators.^1^

## Methods

The stage 1 protocol for this Registered Report was accepted in principle on April 19, 2024 [26]. The protocol, as accepted by the journal, can be found at: 10.6084/m9.figshare.25732482.v1. Any deviations to those preregistered methods are described. See Appendix A for the completed Preferred Reporting Items for Systematic Reviews and Meta Analyses (PRISMA) 2020 Checklist and PRISMA 2020 Checklist for Abstracts, which we used for the writing of this systematic review and meta-analysis [27, 28].

### Eligibility criteria

See Table 1 for inclusion and exclusion criteria. Of note, based on an examination of a subset of 50 records, it became evident that the term “EMA” and its synonyms were sometimes missing from the title and abstract, yet EMA was used as part of the study upon examining the full text. For title and abstract screening, we omitted eligibility criteria regarding study design and outcome to prevent excluding relevant articles. The full eligibility criteria were used for full text screening.

**Table 1:** Inclusion and exclusion criteria for systematic review.

Category	Inclusion Criterion	Exclusion Criterion
Population	• Youth ages 8 to 17 years (inclusive) with psychological symptoms or diagnoses	• Individuals younger than 8 years and older than 17 years without psychological symptoms or diagnoses • Study samples exclusively with medical diagnoses or healthy volunteers • No information on sample size, participant age, or participant psychological symptoms after contacting authors
Study design	• Empirical studies using EMA (synonyms include event and experience sampling methodology; see Appendix B for search strategy with additional synonyms), with survey delivery including text messaging, smartphone applications (“apps”), laptops, and PDAs	• Studies with no EMA methodology, distributing fewer than two surveys per day, or exclusively using paper logs or phone calls to administer surveys • Non-empirical studies
Outcome	• Data provided on enrollment, dropout, and/or compliance	• No information on enrollment, dropout, or compliance after contacting authors
Article characteristics	• English language articles • All geographical locations	• Articles published in a language other than English due to translation limitations of the research team • Preprints, chapters, dissertations, theses, and abstracts due to concerns with peer review • Articles without available full text after checking all NIH access options and contacting authors

*EMA*, ecological momentary assessment, *NIH*, National Institutes of Health, *PDA*, personal digital assistants.

### Information sources

In January 2023, five databases were searched by a biomedical librarian (AAL): Embase (Elsevier), PsycNet: PsycINFO & PsycARTICLES (American Psychological Association), PubMed (US National Library of Medicine), Scopus (Elsevier), and Web of Science: Core Collection (Clarivate Analytics). The searches were limited to English language articles.

To identify other potentially relevant articles, reviewers (LMH, UP, EM, CM, SA, MK, ET, KL, CEH) scanned the bibliographies of included studies and relevant review articles and revisited study protocols flagged during screening to determine if corresponding articles were published prior to the initial search date. In addition, the entire review team, along with relevant unaffiliated collaborators, reviewed the final list of included studies, adding any additional, relevant studies that were missed during initial screening. Articles identified using these supplemental methods proceeded through the screening process outlined below.

### Search strategy

The search strategies were developed by a biomedical librarian (AAL) with expertise in systematic review searches and peer reviewed by a second librarian (NT) not otherwise associated with the project. The review team provided additional feedback on the search terms. The search strategies incorporated keywords and controlled vocabulary terms (i.e., EMTREE [Embase], MeSH [PubMed], Thesaurus of Psychological Index Terms [PsycNet]) for each concept of interest: EMA, digital/mobile devices, psychological symptoms and disorders, and children and adolescents. We used frameworks for the classification of psychopathology (e.g., Hierarchical Taxonomy of Psychopathology; Hi-TOP [29]) to guide search term selection for psychological symptoms and disorders, including relevant spectra (e.g., somatoform, internalizing, thought disorder, externalizing), subfactors (e.g., sexual problems, fear, distress, mania, substance abuse, antisocial behavior), and syndromes/disorders (e.g., bulimia nervosa, posttraumatic stress disorder, oppositional defiant disorder, attention-deficit hyperactivity disorder). We supplemented this list with other terms related to general psychopathology (e.g., psychopatho*, mental disorder*, mental illness*, psychological impair*, behavioral development, behavioral outcome, behavioral wellbeing) and specific symptoms and disorders (e.g., neurodevelopmental disorders, self-harm, irrit*, trauma*). See Appendix B for final search strategies used.

### Study records

#### Data management

EndNote 20 (Clarivate Analytics) was used to collect and manage the results of the literature searches and identify unique articles. Covidence (Veritas Health Innovations), an online tool for systematic review data management, was used to screen the results of the literature searches and for data collection.

#### Selection process

Screening was conducted in two stages. First, titles and abstracts were screened. Second, articles included after title and abstract screening proceeded to full text screening. All reviewers (LMH, UP, EM, CM, SA, MK, ET, KL, CEH) were trained in Covidence prior to screening. Records were double screened (i.e., two reviewers independently screened each record in duplicate) during both stages of screening using the eligibility criteria (Table 1). For both stages, screening discrepancies were resolved during a group consensus meeting. Prior to each stage of screening, a 15-article pilot was conducted in Covidence with all reviewers to refine the eligibility criteria and process with the goal of increasing reliability.

#### Data collection process

We used Covidence for data extraction. Before beginning data extraction, a 5-article pilot was conducted with the purpose of testing the data extraction process and forms. In addition, a 15-article trial was conducted with all reviewers using Covidence with the purpose of increasing reliability.

Reviewers (LMH, EM, CM, ET, KL, CEH, CE, GG, JM, ST, JT) independently extracted key study variables from articles included after full text screening. We coded *k* = 67 (51.54%) studies in duplicate by human reviewers. We estimate that each study took each reviewer 30 min to 1 h to code (including data extraction and quality assessment, described below). We used a commercial large language model (LLM)-based systematic review automation software, Elicit [30], as the second coder for *k* = 63 (48.46%) studies. For human-to-human coding, discrepancies were discussed and resolved via dyadic consensus meetings with the two relevant reviewers for each article. For human-to-software coding, discrepancies were reviewed and resolved by the original human reviewer. Any discrepancies that could not be resolved in this manner were brought to a group consensus meeting and resolved at that time. Use of a commercial LLM-based systematic review automation software was not included in our preregistered methods.

In the case of multiple reports of the same study, we linked relevant reports together prior to data collection using various strategies (e.g., corresponding trial registration numbers, author names, sample characteristics). Then, we extracted data across reports into a single form for use in the review [31]. Authors were contacted by email if a study that otherwise met eligibility criteria was missing information on age or all outcome variables; reports were excluded if the information provided indicated ineligibility, if authors were unable to provide this information, or if we were unable to reach authors.

#### Data items

The following data items were extracted from each included article.

1. Record and study description: authors, year of publication, journal, title, location of study
2. Participant characteristics: sex, age, race and/or ethnicity, receipt of psychotherapy, receipt of psychotropic medication, recruitment status (clinical, community, mixed)
3. Psychopathology: diagnosis or a symptom measure
4. Calculation of primary outcomes (compliance, dropout, enrollment): number of surveys responded to (compliance), number of surveys prescribed (compliance), number of participants completing no surveys (dropout), number of participants completing some surveys but evidencing nonresponse prior to the final data collection day (dropout), number of participants enrolled in the study (dropout, enrollment), number of participants recruited into the study (enrollment)
5. Study design: sampling scheme (e.g., event contingent, fixed, semirandom, random), number of assessment days, number of surveys per day, survey time of day (if fixed or semirandom), time between surveys, types of scales used in surveys, number of items in surveys, average duration of each survey, use of alarms, EMA platform, delivery device (e.g., PDAs, iPhone, Android Phone, laptop, other and personal device, researcher-supplied device, mixed), researcher involvement prior to the study (e.g., coaching, provision of manuals), researcher involvement during study (e.g., research staff contact with participants during the assessment period), individual feedback on compliance, pairing of EMA with other methods, type of compensation, type of study (e.g., intervention), EMA surveys focused on (or including) cognitive testing, parent involvement/support

### Outcomes and prioritization

The primary outcomes of interest were compliance, dropout, and enrollment, in order of prioritization. If outcome statistics were not presented outright, they were calculated. Definitions and formulas for calculating compliance, dropout, and enrollment are provided in Table 2. In short, compliance is the proportion of prescribed surveys that are responded to by participants; dropout (no participation) is the proportion of participants who enrolled but completed no surveys; dropout (some participation) is the proportion of participants who enrolled and completed some surveys but evidenced nonresponse prior to final day of data collection; and enrollment is the proportion of eligible recruited participants who consented to enroll in the study.

**Table 2:** Definitions and formulas for compliance, dropout, and enrollment outcomes.

Outcome	Definition	Formula
Compliance	proportion of prescribed surveys that are responded to by participants	${Compliance}=\frac{{number\; of\; surveys\; responded\; to}}{{number\; of\; surveys\; prescribed}}$
Dropout	proportion of enrolled participants who exhibit nonresponse prior to study completion, calculated two ways	${Dropout},{no\; participation}=\frac{n\,{completing}\,{no}\,{surveys}}{n\,{enrolled}\,{in}\,{the}\,{study}}$ ${Dropout}, {some\; participation}=\frac{{n\; completing}\, {some\; surveys}\; {with\; nonresponse\; prior\; to} \, {study\; completion}}{{n\; enrolled\; in\; the\; study}}$
Enrollment	proportion of recruited participants who consented to enroll in the study	${Enrollment}=\frac{{n\; enrolled\; in\; the\; study}}{{n\; recruited\; into\; the\; study}}$

### Quality assessment in individual studies

As described in previous meta-analyses examining adherence in EMA studies, no standardized risk of bias or quality assessment currently exists for ambulatory studies [6, 20]. Further, the studies included in the current meta-analysis were highly variable, including clinical trials, observational studies, and psychometric studies. Risk of bias or quality of included articles has not been assessed in most previously published EMA adherence meta-analyses [4, 19, 24].

Here, we used an adapted version of the 10-item quality assessment checklist created for Morren et al. [20]’s EMA adherence meta-analysis. The Morren et al. [20] quality assessment tool focuses on determining the level of completeness and accuracy of information in various aspect of each study (e.g., inclusion and exclusion criteria, reliability and validity of measures, statistical analyses).

Before commencing our quality assessment, a 5-article pilot was completed with all reviewers. We assessed *k* = 67 (51.54%) studies in duplicate by human reviewers and used Elicit [30] as the second coder for *k* = 63 (48.46%) studies. Reviewers assessed each article using nine items from Morren et al. [20]’s criteria on a 3-point scale representing poor (0), reasonable (1), and good (2). See Appendix C for items, as well as coding guidance that we developed to support inter-rater reliability for the current study. Final item scores were summed to obtain a total study quality score (range = 0 – 18). For human-to-human coding, discrepancies were discussed and resolved via dyadic consensus meetings with the two relevant reviewers for each article. For human-to-software coding, discrepancies were reviewed and resolved by the original human reviewer. Any discrepancies that could not be resolved in this manner were brought to a group consensus meeting and resolved at that time.

### Data synthesis

#### Systematic review

We provided descriptive statistics on key variables collected (see Data Items) and presented this information in narrative summary format, as well as in key characteristics summary tables and other tables specific to variables and outcomes of interest.

#### Meta-analysis

We conducted meta-analysis in the full sample of included studies and then separately by group. Guided by frameworks for the classification of psychopathology (e.g., Hi-TOP [29]), we (LMH, KL) sorted study samples into one of nine groups representing samples of youth with psychopathology (externalizing, internalizing, neurodevelopmental, trauma exposure/at risk, eating pathology, somatic symptoms, and thought disorder)^2^, healthy samples, and community samples. To be included in a psychopathology group, 75% or more of youth in a study met criteria for disorders/symptoms representative of that group. In some cases, studies included youth in multiple groups (e.g., discrete subsamples of youth with anxiety disorders and attention-deficit/hyperactivity disorder) with unique outcome statistics; in these cases, each subsample was sorted into its corresponding group (e.g., internalizing, neurodevelopmental). Youth in the healthy group were reported as free from psychiatric disorders. Usually, these youth represented a typically developing comparison group in studies of youth with psychopathology. Youth in the community group were typically part of clinical research studies examining continuous symptoms (e.g., depression symptoms via the Beck Depression Inventory-Second Edition [32]). These groups were developed after our analyses were preregistered.

We conducted analyses using R Statistical Software [33] and the metafor package [34]. We used random-effects models (anticipating considerable between-study heterogeneity) with restricted maximum likelihood estimation [35]. All adherence outcomes (compliance, dropout, enrollment) were proportions requiring transformation prior to pooling. We logit transformed compliance proportions using the equation log_e_[*p*/(1-*p*)], where *p* was the proportion. We estimated the standard errors using the equation √[(1/*np*) + [1/*n*(1–*p*)]], where *n* was the sample size and *p* was the proportion [19, 24]. Considering the nested designs of EMA studies (i.e., prompts within participants), we calculated *n* as the effective sample size [36]. For the intraclass correlation coefficient, we were able to extract standard deviations for compliance from 27 studies, yielding minimum (0.06), median (0.16), and maximum (0.43) values. Like previous research [19, 24], minimum, median, and maximum values did not appreciably impact results in sensitivity analyses and so results using the median are presented below. A number of proportions for dropout and enrollment were 0 and 1, respectively, yielding undefined values under logit transformation. To avoid excluding these studies from analyses or applying a constant continuity correction, we arcsine transformed dropout and enrollment [37, 38]. Meta-analysis was conducted when *k* ≥ 3.

Following Jones et al. [19], we derived a pooled estimate of compliance in each group and compared those estimates to the benchmark for adequate EMA survey compliance of 80%. Given that there is no benchmark for enrollment and dropout, we did not complete this step for enrollment and dropout.

We tested for outliers in each meta-analysis model. We examined extreme values in the distribution (i.e., absolute value of standardized residuals > 2), as well as effect sizes with confidence intervals that did not overlap with any other individual confidence interval or the confidence interval for the pooled estimate. For cases determined to be influential, we conducted analyses with those cases included and excluded. Conditional upon sufficient heterogeneity and *k* ≥ 10, we examined participant characteristics (sex, age) and study quality as moderators for each adherence model (compliance, dropout, enrollment) and, additionally, we examined aspects of study design as moderators for each compliance model. We used the *I*^2^ statistic to quantify heterogeneity across effect sizes, and we used the *Q* statistic to test heterogeneity reduction through inclusion of moderators.

### Meta-biases

Trim and fill analyses were conducted on observed outcomes to examine symmetry around pooled estimates to determine missing effects that would impact those outcomes [39]. Publication bias was examined using Egger’s test for funnel plot asymmetry [40].

## Results

### Systematic review

#### Study selection

**Fig. 1:**
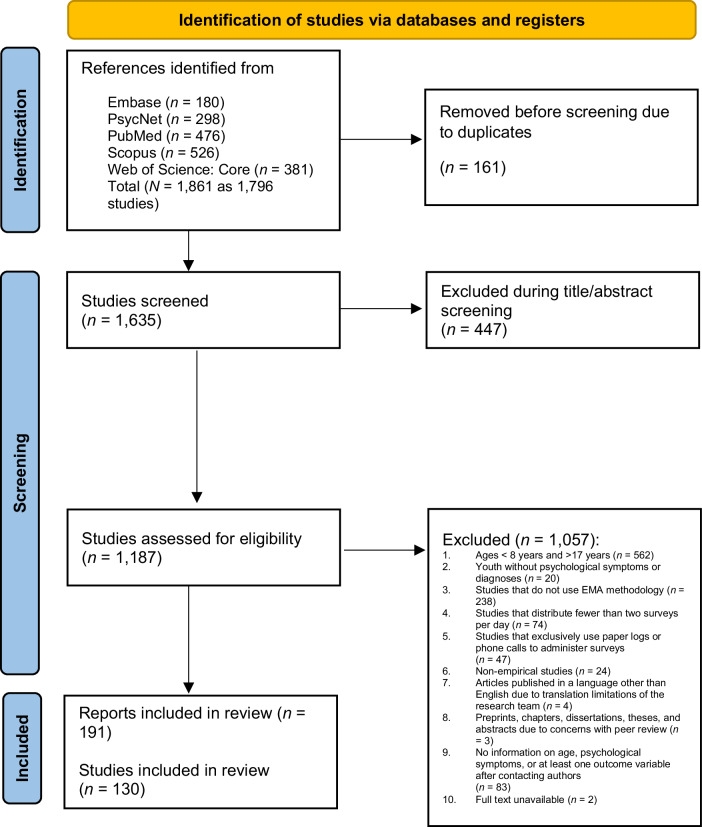
Preferred Reporting Items for Systematic Reviews and Meta Analyses (PRISMA) flow diagram for the current systematic review.

We identified 1,861 references across the database searches and supplemental methods used. After removing duplicates, 1,635 studies were title and abstract screened; we excluded 447 studies and retrieved 1,187 full texts. We excluded 1,057 studies through full-text screening, yielding 130 studies (via 191 records) included in the review. Note that 61 records were merged as they reported the data from a single study. See Fig. 1 for the corresponding PRISMA flow diagram [27, 28].

Inter-rater reliability was acceptable for all stages of the review process. We recorded and summarized inter-rater reliability from Covidence for title and abstract screening (Cohen’s Kappa *M* = 0.75, 95% CI = 0.64 – 0.86) and full text screening (Cohen’s Kappa *M* = 0.87, 95% CI = 0.79 – 0.94) [41], and we calculated inter-rater reliability manually for data extraction (human-to-human percent agreement *M* = 87.90%, *SD* = 5.96, range = 73.30 – 97.16; human-to-software percent agreement *M* = 81.83%, *SD* = 7.96, range = 59.09 – 94.89) and quality assessment (human-to-human percent agreement *M* = 72.59%, *SD* = 14.76, range = 33.33 – 100.00; human-to-software percent agreement *M* = 70.69%, *SD* = 15.23, range = 33.33 – 100.00).

#### Record and study description

The 191 records included in the review were published in 122 different journals, with *Health Psychology* (*k* = 6) being the most common. Records were published between 2001 and 2023. Studies were most frequently conducted in the United States (54.62%, *k* = 71) followed by Germany (11.54%, *k* = 15), the Netherlands (7.69%, *k* = 10), and Australia (7.69%, *k* =10). A subset of studies (13.85%, *k* = 18) collected EMA data in the context of intervention research. See Supplementary Table 1 for full included record and study description characteristics (authors, year of publication, journal, title, location of study, intervention study) for the review.

#### Participant characteristics

There were 14,400 participants across all 130 studies. On average, study sample size totaled 111 participants (*SD* = 164.77) with between 2 and 1,195 participants per study. Omitting community (*n* = 10,357) and healthy (*n* = 528) subsamples, there were 3,515 youth with psychopathology. Most studies (*k* = 57) reported 50–74% of their sample as female, followed by 75–100% as female (*k* = 33), 25–49% as female (*k* = 28), and 0–24% as female (*k* = 10). Two studies did not report on the portion of their sample that was female. Most studies reported 50–74% (*k* = 28) or 75–100% (*k* = 25) of their sample as white, followed by 0–24% (*k* = 11) and 25–49% (*k* = 8). Eight percent (8.46%, *k* = 11) of studies focused on children 8–10 years, 5.38% (*k* = 7) 11–12 years, 33.08% (*k* = 43) 13–15 years, 26.15% (*k* = 34) 16–18 years, and 26.92% (*k* = 35) 19+ years.^3^ Of the 26 studies with data on participant receipt of psychotherapy, 23 (88.46%) reported that at least some participants were receiving psychotherapy. Of the 37 studies with data on psychotropic medication use, 20 (54.05%) reported that at least some participants were using psychotropic medication. See Supplementary Table 2 for participant characteristics by study.

#### Psychopathology

We sorted sixty studies (or subsamples) into psychopathology groups: externalizing (*k* = 13), internalizing (*k* = 12), neurodevelopmental (*k* = 13), trauma exposure/at risk (*k* = 4), eating pathology (*k* = 8), somatic symptoms (*k* = 5), and thought disorder (*k* = 2). Three studies could not be sorted into higher-order categories (e.g., participants had various diagnoses) but were eligible for inclusion in omnibus (full sample, psychopathology collapsed) analyses. Of note, two studies included youth in multiple psychopathology groups with unique outcome statistics and were sorted into multiple different groups. Importantly, many articles reported that at least a subset of their clinical sample had comorbid psychiatric disorders.

In addition to reporting psychiatric diagnoses, many studies included measures of psychological symptoms. The most frequently used measures included the Positive and Negative Affect Scale positive subscale (*k* = 11) and negative subscale (*k* = 10) [42], the Beck Depression Inventory- Second Edition (*k* = 9) [32], EMA items capturing negative affect (*k* = 9) and positive affect (*k* = 8), the Center for Epidemiological Studies Depression Scale (*k* = 6) [43], the Patient Health Questionnaire-9 (*k* = 5) [44], and the Children’s Depression Inventory (*k* = 4) [45]. See Supplementary Table 3 for information on psychopathology by study.

#### Study design

##### Study characteristics

On average, EMA data was collected for 2 weeks (*M* = 13.93 days, *SD* = 18.12, range = 1 – 182) with 6 prompts per day (*M* = 5.96, *SD* = 3.99, range = 1.5 – 27.5). Some studies reported collecting other ambulatory data alongside EMA data (13.85%, *k* = 18). Of those that did, the most frequently implemented methods were accelerometry and actigraphy (50.00%, *k* = 9). Studies also collected phone usage data (e.g., call and text logs, screen locks, light sensors; *k* = 3) and GPS coordinates (*k* = 1), conducted ambulatory saliva sampling (*k* = 2) and blood pressure monitoring (*k* = 1), and implemented a combination of methods using various devices (*k* = 2). See Supplementary Table 4a for study characteristics by study.

##### Survey design

On average, surveys contained 15 items (*M* = 14.68, *SD* = 11.69, range = 1 – 60) and took over 3 min to complete (*M* = 3.53, *SD* = 3.12, range = 0.5 – 16). Most studies reported the types of scales used in their surveys (96.92%, *k* = 126). Of those studies, many incorporated multiple scale types (60.32%, *k* = 76). The most common scale used was the Likert scale (*k* = 88), followed by multiple choice – single choice (*k* = 46), yes/no questions (*k* = 39), visual analogue scale (*k* = 33), multiple choice – select multiple (*k* = 23), and free response (*k* = 15). Two studies reported integrating cognitive testing into their design. See Supplementary Table 4a for survey design by study.

##### Sampling schemes

In terms of survey scheduling, implementing a single sampling scheme was most common (78.46%, *k* = 102), though some studies incorporated multiple schemes (21.54%, *k* = 28). The semirandom sampling scheme was implemented in 52.31% of studies (*k* = 68), followed by fixed (26.15%, *k* = 34), random (23.85%, *k* = 31), event-contingent (16.92%, *k* = 22), and user-initiated (1.54%, *k* = 2) schemes. Across schemes, time between surveys ranged widely from 30 min to 12 h. Forty-nine studies reported survey alarms with delay and/or reminder features.

Studies with semirandom schemes used 5 blocks on average (*M* = 5.46, *SD* = 3.92), and 1 was the modal number of surveys per block. In some studies, block times varied between weekdays and weekends; in others they did not. In some studies, participants selected block times, and in others block times were consistent across participants. Several studies scheduled blocks around school (e.g., after waking/before school, after school, before bedtime).

Fixed schemes deployed surveys as early as 7:00 AM and as late as 11:00 PM. The number of fixed surveys varied across studies. Examples include one survey upon waking (in these cases, the studies also employed another scheme type, otherwise they would be excluded from the current review; see Table 1); one survey distributed in the morning, afternoon, and evening; and one survey every 30 min during waking hours. See Supplementary Table 4b for sampling scheme by study.

##### Devices and platforms

Typically, studies reported delivering surveys using a smartphone or cell phone (64.62%, *k* = 84). Personal digital assistants (PDAs) or handheld personal computers (PCs; 26.15%, *k* = 34), iPod touches (1.54%, *k* = 2), watches (1.54%, *k* = 2), and combinations of various types of devices (e.g., smartphones, tablets, iPod touches, laptops; 3.08%, *k* = 4) were also used. In four studies, the device type was not reported (3.08%, *k* = 4). Devices were supplied by researchers (58.46%, *k* = 76), participants’ personal devices (21.54%, *k* = 28), a mixture of both cases (14.62%, *k* = 19), or not reported (5.38%, *k* = 7). Of the studies that reported the type of platform used (70.00%, *k* = 91), a notable portion was developed for the study (26.37%, *k* = 24). Numerous different platforms were used, with the most common being Movisens (*k* = 9) and Metricwire (*k* = 7). See Supplementary Table 4b for devices and platforms by study.

##### Researcher training and feedback

Researcher interactions with participants varied across studies. Prior to the assessment period, sixty-three studies (48.46%) reported some type of training, such as practice sessions to instruct participants on the use the devices/platforms and training in items and scales. Nine studies explicitly stated that participants were provided with instructional manuals or materials. Nineteen studies reported that onboarding occurred in person, and three studies reported virtual onboarding. During the assessment period, researchers reported contacting participants by phone (*k* = 21), in person (*k* = 9), digitally (e.g., email, messaging) (*k* = 8), through multiple methods (*k* = 6), or using another undefined method (*k* = 11).

The majority of studies did not report whether participants received individual feedback on their compliance (78.46%, *k* = 102). Of those that did (*k* = 28), the majority of the feedback was broadly described as being delivered by research assistants (*k* = 22). In other studies, feedback was delivered digitally (*k* = 4; e.g., through the EMA platform, emails to parents, or text messages). One study reported closely monitoring compliance via the EMA platform but did not report the feedback method. One study explicitly stated that feedback on compliance was not provided to participants.

##### Parental support and involvement

Eleven studies (8.46%) reported on parental support and involvement. In 5 studies, parents were involved by completing EMAs about themselves, their children, or both. Six studies reported that parents were involved in another way, including by children using the parent’s phone to complete EMA, keeping track/being notified of their child’s compliance/participation, reminding/supporting their child with another ambulatory aspect of the study (i.e., saliva sampling), or training on the EMA survey items alongside their children so they might offer support, as necessary. See Supplementary Table 4c for researcher support and parental support by study.

##### Compensation

Of the studies that reported participant compensation (72.31%, *k* = 94), strategies included incentive structures (58.51%, *k* = 55), fixed payments or class credit (28.72%, *k* = 27), no compensation (6.38%, *k* = 6), and gifts (6.38%, *k* = 6). In some studies, compensation differed by participant type (e.g., university students received class credit while participants recruited via advertisements received a fixed payment). In some studies, participants could select compensation preference among options (e.g., course credit or incentive structure). See Supplementary Table 4c for compensation by study.

#### Adherence (primary outcomes)

We operationalized compliance as the percent of prescribed surveys responded to by participants. In 119 studies (91.54%), we could either calculate compliance or compliance was reported. We calculated dropout in two ways. First, we calculated dropout as the percent of participants who enrolled but completed no surveys (“dropout no participation”). This dropout metric was reported, or there was enough information to calculate it, in approximately half (*k* = 64, 49.23%) of the included studies. Second, we calculated dropout as the percent of participants who enrolled and completed some surveys, but evidenced nonresponse prior to final day of data collection.^4^ Again, dropout some was reported or calculated in approximately half (*k* = 62, 47.69%) of the included studies. We operationalized enrollment as the percent of recruited participants who consented to enroll in the study. Enrollment was reported or calculated in approximately one third (*k* = 43, 33.08%) of the included studies. See Supplementary Table 3 for adherence by study.

#### Quality assessment

We adapted Morren et al. [20]’s checklist for our quality assessment. On average, studies received a total score of 14.01 (*SD* = 2.33, range = 6–18), which could be considered moderate-to-high given the possible range of 0 to 18. All individual items received scores spanning the full scale (0 = *poor*, 1 = *reasonable*, 2 = *good*) except “Design” and “Research Questions Answered,” on which no studies received a score of poor. See Supplementary Table 3 for quality assessment by study.

### Meta-analyses

See Table 3 for a summary of meta-analytic results for each outcome, including heterogeneity statistics (*Q*, *I*^2^).

**Table 3:** Meta-analytic results by outcome.

Group/Moderator		*k*^a^	Outcome (%) /Moderator effect^a^ (95% CI)	Significantly different from 80% compliance and direction?	*Q* for Group or Moderator	*I*^*2*^ (%)
**Compliance**						
**Full Sample**		119	74.39 (71.49, 77.08)	Yes, lower	2,558.93, *p* < 0.001	93.19
*3 extreme values removed*		116	74.93 (72.37, 77.33)	Yes, lower	1,074.37, *p* < 0.001	90.67
Scales (ref: Likert)	VAS	113	0.54 (0.06, 1.03)	NA	17.12, *p* = 0.004	89.62
Compensation (ref: Incentive)	Flat fee^b^	86	−0.51 (−0.82, −0.20)	NA	17.50, *p* < 0.001	88.12
	Gift		−0.91 (−1.57, −0.24)			
	None		−0.65 (−1.23, −0.06)			
**Community**		67	73.60 (69.54, 77.29)	Yes, lower	2,023.35, *p* < 0.001	94.84
*2 extreme values removed*		65	73.87 (70.25, 77.20)	Yes, lower	729.70, *p* < 0.001	92.78
Scales (ref: Likert)	VAS	64	0.63 (0.01, 1.25)	NA	18.71, *p* = 0.002	90.99
	Multiple		−0.41 (−0.79, −0.04)			
Compensation (ref: Incentive)	Flat fee^b^	49	−0.66 (−1.03, −0.28)	NA	18.40, *p* < 0.001	88.15
	Gift		−0.88 (−1.56, −0.21)			
	None		−1.19 (−2.08, −0.30)			
**Healthy**^**c**^		14	80.32 (75.14, 84.64)	No	29.78, *p* = 0.005	58.03
*1 extreme value removed*		13	78.31 (73.76, 82.26)	No	19.91, *p* = 0.07	37.82
Alarms (ref: Not reported)		13	0.58 (0.13, 1.04)	NA	6.32, *p* < 0.01	8.28
**All Psychopathology**		53	74.43 (70.03, 78.39)	Yes, lower	438.41, *p* < 0.001	87.24
*1 extreme value removed*		52	75.38 (71.54, 78.86)	Yes, lower	308.04, *p* < 0.001	83.19
Age		52	−0.06 (−0.10, −0.02)	NA	7.52, *p* = 0.006	80.58
Reminders (ref: Not reported)		52	0.85 (0.15, 1.56)	NA	5.58, *p* = 0.02	81.43
**Internalizing**^**a**^		10	75.61 (67.86, 81.98)	No	41.41, *p* < 0.001	77.80
*1 extreme value removed*		9	77.69 (71.59, 82.79)	No	21.96, *p* = 0.005	63.05
**Externalizing**		13	72.29 (62.47, 80.35)	No	151.91, *p* < 0.001	93.06
% female		13	−0.03 (−0.05, −0.01)	NA	8.88, *p* = 0.003	87.47
Sampling scheme (ref: Semirandom)	Multiple	13	1.07 (0.25, 1.89)	NA	9.92, *p* = 0.02	85.48
	Fixed		1.37 (0.33, 2.40)			
**Neurodevelopmental**^**c**^		13	78.60 (73.25, 83.12)	No	16.34, *p* = 0.18	30.68
Alarms (ref: Not reported)		13	0.82 (0.34, 1.30)	NA	11.13, *p* < 0.001	0.00
Reminders (ref: Not reported)		13	1.10 (0.41, 1.80)	NA	9.77, *p* = 0.002	0.00
**Eating Pathology**^a^		8	75.01 (66.96, 81.63)	No	20.45, *p* = 0.005	64.83
**Thought Disorders**^**a**^		2	NA	NA	NA	NA
**Trauma Exposure/At Risk**^**a**^		3	76.44 (47.21, 92.17)	No	41.64, *p* < 0.001	96.04
**Somatic Symptoms**^**a**^		5	77.38 (62.98, 87.31)	No	14.48, *p* = 0.006	70.85

Dropout (No Participation)						
**Full Sample**		64	2.22 (1.04, 3.84)	NA	3,317.09, *p* < 0.001	93.15
*1 extreme value removed*		63	1.80 (0.94, 2.94)	NA	442.28, *p* < 0.001	86.65
**Community**		31	1.52 (0.52, 3.03)	NA	270.13, *p* < 0.001	89.02
**Healthy**		7	0.41 (0.00, 2.05)	NA	7.84, *p* = 0.25	33.27
**All Psychopathology**		34	3.16 (0.99, 6.50)	NA	2,271.89, *p* < 0.001	93.60
*1 extreme value removed*		33	2.26 (0.85, 4.32)	NA	169.90, *p* < 0.001	82.55
**Internalizing**		4	1.24 (0.00, 5.11)	NA	8.01, *p* = 0.05	62.75
**Externalizing**		7	10.33 (0.76, 28.87)	NA	912.31, *p* < 0.001	98.25
*1 extreme value removed*		6	4.64 (0.05, 12.46)	NA	60.85, *p* < 0.001	89.48
**Neurodevelopmental**		9	1.91 (0.00, 7.27)	NA	25.04, *p* = 0.002	69.89
**Eating Pathology**		4	4.51 (0.12, 14.72)	NA	21.70, *p* < 0.001	84.23
**Thought Disorders**		2	NA	NA	NA	NA
**Trauma Exposure/At Risk**		2	NA	NA	NA	NA
**Somatic Symptoms**		5	0.29 (0.00, 1.72)	NA	2.11, *p* = 0.72	0.46

Dropout (Some Participation)						
**Full Sample**		62	8.54 (6.56, 10.74)	NA	630.31, *p* < 0.001	86.82
**Community**		38	7.74 (5.63, 10.17)	NA	469.90, *p* < 0.001	86.62
**Healthy**		5	3.24 (0.26, 9.37)	NA	8.19, *p* = 0.08	54.44
**All Psychopathology**		23	11.55 (7.16, 16.83)	NA	130.98, *p* < 0.001	83.57
**Internalizing**		6	12.70 (5.00, 23.26)	NA	35.39, *p* < 0.001	85.37
**Externalizing**		4	16.65 (6.91, 29.55)	NA	19.81, *p* < 0.001	87.69
**Neurodevelopmental**		6	6.09 (0.00, 24.77)	NA	56.02, *p* < 0.001	89.78
*1 extreme value removed*		5	1.85 (0.00, 7.71)	NA	7.99, *p* = 0.09	51.04
**Eating Pathology**		2	NA	NA	NA	NA
**Thought Disorders**		2	NA	NA	NA	NA
**Trauma Exposure/At Risk**		1	NA	NA	NA	NA
**Somatic Symptoms**		4	5.12 (1.45, 10.86)	NA	2.93, *p* = 0.40	1.59

Enrollment						
**Full Sample**		44	71.21 (62.93, 78.82)	NA	3,803.58, *p* < 0.001	99.02
Age		43	−0.02 (−0.04, −0.003)	NA	5.24, *p* = 0.02	98.84
**Community**		25	64.65 (54.53, 74.14)	NA	1,435.54, *p* < 0.001	98.74
*1 extreme value removed*		24	67.07 (57.99, 75.55)	NA	1,152.51, *p* < 0.001	98.41
**Healthy**		0	NA	NA	NA	NA
**All Psychopathology**		21	76.01 (62.12, 87.55)	NA	2,110.78, *p* < 0.001	98.97
*2 extreme values removed*		19	82.17 (71.84, 90.54)	NA	1,467.86, *p* < 0.001	97.56
**Internalizing**		2	NA	NA	NA	NA
**Externalizing**		7	61.80 (35.83, 84.58)	NA	914.48, *p* < 0.001	99.49
*1 extreme value removed*		6	69.91 (46.36, 88.91)	NA	623.52, *p* < 0.001	98.75
**Neurodevelopmental**		2	NA	NA	NA	NA
**Eating Pathology**		2	NA	NA	NA	NA
**Thought Disorders**		2	NA	NA	NA	NA
**Trauma Exposure/At Risk**		1	NA	NA	NA	NA
**Somatic Symptoms**		3	47.09 (6.21, 90.69)	NA	206.47, *p* < 0.001	98.77

Meta-analyses were not conducted for *k* < 3 and moderator analyses were not conducted for *k* < 10 to support robust, reliable results.

*VAS*, visual analogue scale. *NA*, Not Applicable. *Ref*, reference variable.

^a^Moderator effect is represented as Δ logit vs. reference.

^b^Flat fee includes both course credit and monetary compensation without an incentive structure.

^c^Examination of moderators was not warranted considering heterogeneity statistics. We examined age, %female, and study quality separately as covariates with nonsignificant effects.

#### Compliance

##### Full sample

Across 119 effect sizes (community, healthy, and psychopathology youth), the overall compliance rate was 74.39% (95% CI = 71.49, 77.08%) – significantly lower than the 80% compliance benchmark for adequate EMA survey compliance. After removing three extreme cases, compliance was 74.93% (95% CI = 72.37, 77.33%). Compliance was moderated by type of survey scale and compensation. We observed greater compliance in studies with visual analogue compared to Likert scales (reference group). Studies providing participants with gifts, flat fees or course credit, and no reimbursement had lower compliance than studies with incentive structures (reference group).

##### Community and healthy

The compliance rate for the community sample was 73.60% (67 effect sizes, 95% CI = 69.54%, 77.29%) – significantly lower than the 80% compliance benchmark. After removing two extreme cases, compliance was 73.87% (95% CI = 70.25%, 77.20%). We observed higher compliance in studies with visual analogue scales, and lower compliance in studies with multiple scales, compared to Likert scales (reference group). Studies providing participants with gifts, flat fees or course credit, and no reimbursement had lower compliance than studies with incentive structures (reference group). The compliance rate for the healthy sample was 80.32% (14 effect sizes, 95% CI = 75.14%, 84.64%) – not significantly different than the 80% compliance benchmark. After removing one extreme case, compliance was 78.31% (95% CI = 73.76%, 82.26%). We observed greater compliance in studies that used alarms (compared to studies that did not report using alarms).

##### Psychopathology: Collapsed and by group

Across 53 effect sizes, the compliance rate for psychopathology collapsed was 74.43% (95% CI = 70.03%, 78.39%) – significantly lower than the 80% compliance benchmark. After removing one extreme case, compliance was 75.38% (95% CI = 71.54%, 78.86%). We observed lower compliance in studies with participants older in age. We observed greater compliance in studies deploying survey reminders (compared to studies that did not report deploying survey reminders). Compliance was not significantly different than the 80% benchmark for externalizing (13 effect sizes, 72.29% [95% CI = 62.47%, 80.35%]), internalizing (9 effect sizes with one extreme value removed, 77.69% [95% CI = 71.59%, 82.79%]), eating pathology (8 effect sizes, 75.01% [95% CI = 66.96%, 81.63%]), neurodevelopmental (13 effect sizes, 78.60% [95% CI = 73.25%, 83.12%]), somatic symptoms (5 effect sizes, 77.38% [95% CI = 62.98%, 87.31%]), and trauma exposure/at risk (3 effect sizes, 76.44% [95% CI = 47.21%, 92.17%]). For externalizing, we observed lower compliance in studies with higher proportions of females. We observed higher compliance in studies with mixed and fixed sampling schemes compared to semirandom schemes (reference group). For neurodevelopmental, we observed greater compliance in studies that used alarms and reminders (compared to studies that did not report using alarms and reminders). Due to too few effect sizes, we were unable to conduct meta-analyses for the thought disorders group.

#### Dropout (no participation)

##### Full sample

Across 64 effect sizes, dropout for the full sample was 2.22% (95% CI = 1.04%, 3.84%). After removing one extreme case, dropout was 1.80% (95% CI = 0.94%, 2.94%).

##### Community and healthy

Dropout was 1.52% for the community sample (31 effect sizes, 95% CI = 0.52%, 3.03%) and 0.41% for the healthy sample (7 effect sizes, 95% CI = 0.00%, 2.05%).

##### Psychopathology: Collapsed and by group

Across 34 effect sizes, dropout for psychopathology collapsed was 3.16% (95% CI = 0.99%, 6.50%), which reduced to 2.26% (95% CI = 0.85%, 4.32%) after removing one extreme case. By group, dropout was 1.24% for internalizing (4 effect sizes, 95% CI = 0.00%, 5.11%), 4.64% for externalizing after removing one extreme case (6 effect sizes, 95% CI = 0.05%, 12.46%]), 1.91% for neurodevelopmental (9 effect sizes, 95% CI = 0.00%, 7.27%]), 4.51% for eating pathology (4 effect sizes, 95% CI = 0.12%, 14.72%), and 0.29% for somatic symptoms (5 effect sizes, 95% CI = 0.00%, 1.72%). Effect sizes for the healthy sample and the internalizing, neurodevelopmental, and somatic symptoms groups were not significantly different than 0%. There were too few effect sizes for thought disorders and trauma exposure/at risk to conduct meta-analyses.

#### Dropout (some participation)

##### Full sample

Across 62 effect sizes, dropout for the full sample was 8.54% (95% CI = 6.56%, 10.74%).

##### Community and healthy

Dropout was 7.74% for the community sample (38 effect sizes, 95% CI = 5.63%, 10.17%) and 3.24% for the healthy sample (5 effect sizes, 95% CI = 0.26%, 9.37%).

##### Psychopathology: Collapsed and by group

Across 23 effect sizes, dropout for psychopathology collapsed was 11.55% (95% CI = 7.16%, 16.83%). By group, dropout was 12.70% for internalizing (6 effect sizes, 95% CI = 5.00%, 23.26%), 16.65% for externalizing (4 effect sizes, 95% CI = 6.91%, 29.55%), 1.85% for neurodevelopmental (5 effect sizes with one extreme case removed, 95% CI = 0.00%, 7.71%], and 5.12% for somatic symptoms (4 effect sizes, 95% CI = 1.45%, 10.86%). There were too few effect sizes for eating pathology, thought disorders, and trauma exposure/at risk to conduct meta-analyses.

#### Enrollment

##### Full sample

Across 44 effect sizes, enrollment for the full sample was 71.21% (95% CI = 62.93%, 78.82%). We observed greater enrollment in studies with participants younger in age.

##### Community and healthy

Enrollment was 64.65% for the community sample (25 effect sizes, 95% CI = 54.53%, 74.14%). After removing one extreme case, enrollment was 67.07% (95% CI = 57.99%, 75.55%). There were no effect sizes for the healthy sample, and so we were unable to conduct meta-analyses.

##### Psychopathology: Collapsed and by group

Across 21 effect sizes, enrollment for psychopathology collapsed was 76.01% (95% CI = 62.12%, 87.55%), which increased to 82.17% (95% CI = 71.84%, 90.54%) with two extreme cases removed. By group, enrollment was 69.91% for externalizing (6 effect sizes with one extreme value removed, 95% CI = 46.36%, 88.91%]) and 47.09% for somatic symptoms (3 effect sizes, 95% CI = 6.21%, 90.69%). We were unable to conduct meta-analyses for internalizing, neurodevelopmental, eating pathology, thought disorders, and trauma exposure/at risk groups due to too few effect sizes.

### Meta-biases

See Supplementary Table 5 for a summary of results from trim and fill analyses and Egger’s regression tests. See Appendix D for funnel plots.

#### Compliance

Trim and fill analyses identified missing studies for the full sample (*k* = 22), community (*k* = 9), healthy (*k* = 5), and psychopathology collapsed (*k* = 10). Two missing studies were identified for  the internalizing group, and one missing study was identified for the eating pathology and somatic symptoms groups. After imputing hypothetical missing studies, compliance rates were reduced in all cases. For the most part, conclusions did not change. The full sample, community sample, and psychopathology collapsed remained lower than the 80% benchmark for adequate EMA survey compliance. The internalizing, eating pathology, and somatic symptoms groups remained no different than the benchmark. For the healthy sample, however, the pooled effect became significantly lower than the 80% benchmark. Egger’s regression tests provided evidence of small study effects for the full, community, healthy, and internalizing groups. Meta-biases could not be assessed for the thought disorders group due to too few effect sizes.

#### Dropout (no participation)

Trim and fill analyses identified missing studies for the full sample (*k* = 5), community sample (*k* = 2), psychopathology collapsed (*k* = 4), internalizing (*k* = 1), neurodevelopmental (*k* = 1), and somatic symptoms (*k* = 1). After imputing hypothetical missing studies, dropout rates increased for all groups. Egger’s regression tests did not provide evidence of small study effects for any group.

#### Dropout (some participation)

Trim and fill analyses identified missing studies for the full sample (*k* = 4), community sample (*k* = 12), healthy (*k* = 1), psychopathology collapsed (*k* = 2), neurodevelopmental (*k* = 2), and somatic symptoms (*k* = 1). After imputing hypothetical missing studies, dropout rates increased for the full sample, healthy sample, psychopathology collapsed, and neurodevelopmental and somatic symptoms groups. Dropout rates decreased for the community sample. Egger’s regression tests provided evidence of small study effects for the externalizing group.

#### Enrollment

Trim and fill analyses identified missing studies for the full sample (*k* = 5), psychopathology collapsed (*k* = 3), and the externalizing group. In all cases, enrollment decreased after imputing hypothetical missing studies. Egger’s regression tests provided evidence of small study effects for the full sample.

## Discussion

### Overview

EMA is a tool on the rise in clinical research for naturalistic, real-time, and repeated data collection. We conducted a systematic review with meta-analysis to support clinical researchers in optimizing their EMA protocols. Specifically, we provided an overview of EMA protocols in clinical pediatric research, and we (1) examined EMA enrollment, dropout, and compliance in children and adolescents with a wide range of psychological disorders and symptoms and (2) identified aspects of study design that maximize adherence.

### Systematic review and meta-analysis

Our systematic review yielded 130 included studies with 14,400 participants. On average, surveys contained 15 items and took over 3 min to complete. Many studies incorporated multiple item response scale types – commonly the Likert scale. Surveys were typically delivered through a single sampling scheme, such as semirandom, random, fixed, or event contingent. Over half of studies reported delivering surveys by smartphone/cell phone and providing those devices to participants. Participants were most commonly compensated using incentive structures.

We found reporting of EMA study enrollment to be low (33%) but higher than in previous reviews of adult research (i.e., 28%, [20]). Similarly, we found enrollment itself to be higher in our pediatric samples (71% overall; 82% in youth with psychopathology and 67% in community youth) than in previous research with adult samples (i.e., 53%, [20]). Higher enrollment in child compared to adult EMA studies may be driven by a variety of factors, such as the parent’s role in seeking out and following through with the research process. This is underscored by our finding of greater enrollment in studies with participants younger in age in the full sample. Greater observed enrollment in children with psychopathology than community samples may be related to a motivation to seek out and follow through with research that can promote recovery.

Like enrollment, reporting of EMA study dropout was low (half of all included studies). Rates of dropout (no participation) – or enrolling and completing no surveys – were relatively low and comparable across youth with psychopathology (2%), community youth (2%), and healthy youth (<1%). Rates of dropout (some participation) – or completing some surveys but showing nonresponse prior to the final day of data collection – were greatest in youth with psychopathology (12%), followed by community youth (8%) and healthy youth (3%). Results provide nuance to previous dropout estimates that range widely (e.g., from 7% [6] to 19% [20]). That is, while researchers may excel in obtaining buy-in from youth with psychopathology and community youth such that they initiate EMA protocols, researchers and participants may need more support in maintaining engagement throughout the EMA protocol.

Previous meta-analytic research in children and adults has found compliance close to 80% – the field’s benchmark for adequate compliance. Across all studies, compliance was 75%, or significantly lower than that benchmark. Importantly, results varied based on group and study design. Compliance in our sample of healthy youth was no different than the 80% benchmark, while compliance in our sample of community youth was significantly lower (i.e., 74%). Healthy youth were determined to be free of psychiatric diagnoses, whereas diagnostic status was either not evaluated or not reported for community youth. As such, it is possible that psychological disorders and symptoms were present in community youth that contributed to lower compliance. Importantly, compliance was moderated by study design factors for community youth, such that compliance was greater with the inclusion of visual analogue scales and incentive-based compensation structures.

Across studies of youth with psychopathology, compliance was lower than the 80% benchmark (75%). Again, compliance was moderated by participant and study design factors. Regarding participant factors, compliance was greater in studies with younger children. Though we likely did not have enough studies reporting on parent support to observe a moderating effect, it might be that younger children receive more scaffolding (e.g., reminders) than older children which manifests as increased survey compliance. Regarding study design factors, we observed greater compliance in studies deploying survey reminders compared to those that did not. Compliance was not significantly different from 80% in any of the individual psychopathology groups for which we were able to conduct meta-analysis (externalizing, internalizing, eating pathology, neurodevelopmental, somatic symptoms, and trauma exposure/at risk). While noteworthy for clinical researchers looking to include EMA in their studies but hesitant about compliance, results should be interpreted in the context of few studies and wide confidence intervals for some groups (e.g., trauma exposure/at risk group). A number of groups evidenced considerable heterogeneity unexplained by our included moderators. Due to a limited number of studies, we were unable to further decompose groups into diagnostic categories (e.g., neurodevelopmental included youth with autism spectrum disorder and attention-deficit/hyperactivity disorder). As EMA continues to be used in clinical pediatric research, future meta-analyses may parse heterogeneity to further inform study design.

### Strengths

The current research is characterized by strengths related to innovation and stringent methods. First, our systematic review provides a near comprehensive overview of EMA protocols in pediatric clinical research, which may provide researchers with the tools to begin to conceptualize and design their own EMA protocols (see also [1]). Our meta-analysis is the first to report EMA study enrollment for youth with psychopathology; examine EMA study adherence across psychopathology diagnostic groups; and test aspects of study design as moderators of adherence across those groups. Second, we preregistered our methods (with a few deviations that were explicitly reported), and we adhered to the PRISMA 2020 Checklists in conducting our synthesis and reporting our results [27, 28]. Third, we executed a thorough search strategy developed in collaboration with an expert biomedical librarian and peer reviewed by a second librarian yielding 1,636 studies for screening and 131 included studies. Fourth, we double-coded records throughout the selection process (i.e., title/abstract and full-text screening) and during the data collection processes to minimize errors. Fifth, we extracted numerous data items from each included study, including those that to our knowledge have not before been extracted in previous systematic reviews (e.g., parental support), and we conducted quality assessment for each included study.

### Limitations

Our review process was characterized by several limitations that highlight areas for future research. First, our search was expansive. We estimate data extraction and quality assessment alone would have taken over 262 h for our included studies (i.e., two coders at a rate of ~1 h per study for 131 studies), excluding time for dyadic consensus meetings. We used Elicit [30] as the second coder for data extraction and quality assessment for a little over half of our included articles, which reduced our coding time but introduced additional nontrivial time and effort around selecting and learning the software, engineering and piloting the prompts, and obtaining and processing the resulting data. Notably, inter-rater reliability was similar for human-human reviewers and the human-Elicit pairs for both data extraction and quality assessment. Future research warrants careful consideration around artificial intelligence-supported methods for evidence synthesis, including the continued development of guides to support researchers in their use, evaluation of accuracy of various models and software, and ethics. Second, we limited our search to English language and peer-reviewed articles. Focusing only on English language articles may have contributed to bias and influenced the results, such as limiting their generalizability and reducing their robustness. Focusing only on peer-reviewed articles may have contributed to the good quality of our included literature, as evidenced by a moderate-to-high average quality assessment score. Importantly, however, as evidenced through our evaluation of meta-biases, studies were identified as missing which may have been influenced by this eligibility criterion. While imputing the hypothetical missing studies did not substantially change the conclusions of our meta-analyses, the inclusion of gray literature is an important consideration for future reviews. Third, no validated and standardized risk of bias assessment currently exists for ambulatory studies. We used an adapted version of the 10-item quality assessment checklist created for Morren et al.’s [20] EMA adherence meta-analysis, which focuses on level of completeness and accuracy of information across various aspects of each study. Future research would benefit from the development or tailoring of risk of bias assessments for research leveraging emerging technologies, like EMA, other types of ambulatory assessments, and technology-based interventions (e.g., just-in-time adaptive interventions).

There are also limitations of the evidence included in our review. Missing data from our included studies precluded our ability to fully characterize EMA studies and their methods and estimated effects in our meta-analyses. There was a great deal of unexplained heterogeneity despite plans for the comprehensive extraction of design features (moderators). While studies may indicate the presence of a design feature (e.g., feedback on compliance), studies rarely report their absence, which is understandable but interferes with future decision making. Most of the articles were not written with the goal of quantifying adherence and detailing study characteristics. Nevertheless, reporting checklists such as the Checklist for Reporting EMA Methods and Assessments [46] may support researchers in reporting key aspects of EMA studies (e.g., compliance, design) in a standard manner thereby increasing their reproducibility and use in evidence synthesis. In addition, among studies of youth with psychopathology, many reported that at least a subset of their sample had co-morbid psychiatric disorders. This is not surprising, given that psychopathology is by nature comorbid. However, results by group should be interpreted with this real-world context in mind.

## Conclusion

The current research has three key contributions with direct implications for the development and execution of pediatric EMA research protocols. First, our systematic review provided an overview of the design features of EMA studies for pediatric research. Second, results of our meta-analysis underscore the feasibility of enrollment, retention, and survey compliance for EMA studies not only with healthy youth, but also with youth with a range of psychological symptoms and disorders. The importance of maintaining the engagement of youth throughout the EMA study is highlighted. Third, our meta-analysis sheds light on participant characteristics (e.g., younger age) and study characteristics (e.g., incentive-based compensation structures) that may enhance compliance in certain subgroups. These findings can inform future pediatric EMA research protocols toward maximizing enrollment, minimizing dropout, and maximizing compliance.

## Supplementary information

Supplementary Table 1

Supplementary Table 2

Supplementary Table 3

Supplementary Table 4a

Supplementary Table 4b

Supplementary Table 4c

Supplementary Table 5

Appendix A

Appendix B

Appendix C

Appendix D

## Data Availability

This study was pre-registered as Stage 1 Registered Report on April 19, 2024 following in-principle acceptance at: 10.6084/m9.figshare.25732482.v1. The dataset supporting the findings of this study is available in the Open Science Framework (OSF) repository at: https://osf.io/tk5fc/.

## Footnotes

1 In our preregistration, we intended to test our primary and secondary research questions with data stratified by childhood and adolescence. Given that age was a significant moderator of adherence in only one instance, we decided to forgo data stratification to minimize the number of analyses conducted.

2 The externalizing group included youth with substance use or abuse, oppositional-defiant or conduct disorders, and personality disorders. The internalizing group included youth with anxiety disorders, depressive disorders (e.g., major depressive disorder, bipolar I and II), suicide risk, and non-suicidal self injury. The neurodevelopmental group included autism spectrum disorders and attention-deficit/hyperactivity disorder. The trauma exposure/at risk category included trauma exposure and other experiences putting youth at heightened risk for psychopathology. The eating pathology group included anorexia nervosa, bulimia nervosa, binge eating disorder, and other feeding and eating disorders. The somatic symptoms group included youth with chronic or acute health conditions. The thought disorder group included youth with psychotic disorders or symptoms.

3 Studies were included if any participant in the sample met our age inclusion criterion (i.e., 8–17 years, inclusive). Studies were binned using mean age for each study sample. If mean age was not available, median age and then age range were used.

4 In many studies, it was not possible to differentiate participant nonresponse prior to the final day of data collection from overall low participant response. As such, both cases are included in dropout (some participation). This became evident during data collection and after analyses were preregistered.
