# Supplementary material for: Adherence to ecological momentary assessment studies in children and adolescents with psychopathology: A systematic review with meta-analysis
Source: NPP Digit Psychiatry Neurosci. 2026 Apr 27;4:9. doi: 10.1038/s44277-026-00058-z (PMC13121683; doi:10.1038/s44277-026-00058-z)
Supplement: Supplementary file 9 — Appendix B [file 44277_2026_58_MOESM9_ESM.docx]

Appendix B

**Title of Review:** Adherence to ecological momentary assessment studies in children and adolescents with psychopathology: A systematic review with meta-analysis

**Database:** PubMed/MEDLINE
**Platform:** US National Library of Medicine
**Date Searched:** January 11, 2023

**Database Date Coverage:** 1946–present
**Date Limits:** None
**Other Limits/Filters:** Language: English; Publication type: Primary research only (excluding reviews, letters, editorials, conference abstracts/proceedings, dissertations, book, book chapters)

| **Set** | **Concept** | **Search Strategy** |
| --- | --- | --- |
| #1 | Ecological Momentary Assessment | ((Ecological Momentary Assessment[Major MeSH] OR "ecological momentary assessment*"[Title/Abstract] OR “ecological momentary intervention*”[Title/Abstract] OR momentary[Title/Abstract] OR “daily diary”[Title/Abstract] OR “daily diaries”[Title/Abstract] OR “electronic diar*”[Title/Abstract] OR “e diar*”[Title/Abstract] OR “e-diar*”[Title/Abstract] OR ediar*[Title/Abstract] OR “digital diar*”[Title/Abstract] OR "Diaries as Topic"[Mesh] OR "event sampling*"[Title/Abstract] OR "experience sampling*"[Title/Abstract] OR “experience-sampling*”[Title/Abstract] OR "real time assessment*"[Title/Abstract] OR “real-time assessment*”[Title/Abstract] OR “just-in-time assessment*”[Title/Abstract] OR “just in time assessment*”[Title/Abstract] OR "short message service*"[Title/Abstract] OR "ambulatory assessment*"[Title/Abstract] OR "text messag*"[Title/Abstract] OR texting[Title/Abstract] OR "Text Messaging"[Mesh] OR “daily prompt*”[Title/Abstract] OR “electronic prompt*”[Title/Abstract] OR “digital prompt*”[Title/Abstract]) NOT sexting[Title/Abstract]) |
| #2 | Digital/Mobile Devices | (cellphone*[Title/Abstract] OR “cell phone*”[Title/Abstract] OR “cellular phone*”[Title/Abstract] OR “cellular telephone*”[Title/Abstract] OR “mobile telephone*”[Title/Abstract] OR “mobile phone*”[Title/Abstract] OR smartphone*[Title/Abstract] OR “palm pilot*”[Title/Abstract] OR “mobile device*”[Title/Abstract] OR “mobile technolog*”[Title/Abstract] OR app[Title/Abstract] OR apps[Title/Abstract] OR “mobile application*”[Title/Abstract] OR computer[Title/Abstract] OR computers[Title/Abstract] OR tablet*[Title/Abstract] OR iPad*[Title/Abstract] OR laptop*[Title/Abstract] OR iphone*[Title/Abstract] OR iPhone*[Title/Abstract] OR Android[Title/Abstract] OR "Smartphone"[Mesh] OR "Cell Phone"[Mesh] OR "Mobile Applications"[Mesh] OR "Computers, Handheld"[Mesh]) |
| #3 | Psychological Symptoms/Psychopathologies | (somatoform[Title/Abstract] OR “Somatoform Disorders”[Major MeSH] OR "eating disorder*"[Title/Abstract] OR “Feeding and Eating Disorders”[Major MeSH] OR "eating patho*"[Title/Abstract] OR "obsessive compulsive disorder*"[Title/Abstract] OR “Obsessive-Compulsive Disorder”[Majr:Noexp] OR "personality disorder*"[Title/Abstract] OR “Personality Disorders”[Major MeSH] OR "self harm*"[Title/Abstract] OR "self-harm*"[Title/Abstract] OR “Self-Injurious Behavior”[Major MeSH] OR “self-injur*”[Title/Abstract] OR “self injur*”[Title/Abstract] OR automutilat*[Title/Abstract] OR “self mutilate*”[Title/Abstract] OR “self-multilat*”[Title/Abstract] OR “self inflicted injur*”[Title/Abstract] OR "sexual problem*"[Title/Abstract] OR "Sexual Dysfunctions, Psychological"[Majr:Noexp] OR “gender dysphori*”[Title/Abstract] OR “gender identity disorder*”[Title/Abstract] OR “psychosexual disorder*”[Title/Abstract] OR “Sexual and Gender Disorders”[Major MeSH] OR anorex*[Title/Abstract] OR Anorexia[Major MeSH] OR "Anorexia Nervosa"[Major MeSH] OR anxiety[Title/Abstract] OR anxieties[Title/Abstract] OR anxious*[Title/Abstract] OR Anxiety[Major MeSH] OR “Anxiety Disorders”[Major MeSH] OR “binge eat*”[Title/Abstract] OR “binge-eat*”[Title/Abstract] OR “Binge-Eating Disorder”[Major MeSH] OR bipolar[Title/Abstract] OR “Bipolar Disorder”[Major MeSH] OR bulim*[Title/Abstract] OR Bulimia[Major MeSH] OR depressi*[Title/Abstract] OR Depression[Major MeSH] OR “Depressive Disorder”[Major MeSH] OR dysthymi*[Title/Abstract] OR “Dysthymic Disorder”[Major MeSH] OR fear*[Title/Abstract] OR Fear[Major MeSH] OR hypomani*[Title/Abstract] OR Mania[Major MeSH] OR internaliz*[Title/Abstract] OR mania*[Title/Abstract] OR manic[Title/Abstract] OR panic[Title/Abstract] OR panics[Title/Abstract] OR panicked[Title/Abstract] OR Panic[Major MeSH] OR “Panic Disorder”[Major MeSH] OR phobi*[Title/Abstract] OR “Phobic Disorders”[Major MeSH] OR suicid*[Title/Abstract] OR Suicide[Major MeSH] OR trauma*[Title/Abstract] OR "thought disorder*"[Title/Abstract] OR paranoi*[Title/Abstract] OR “Paranoid Disorders”[Major MeSH] OR psychosis[Title/Abstract] OR psychotic[Title/Abstract] OR “Psychotic Disorders”[Major MeSH] OR schizo*[Title/Abstract] OR Schizophrenia[Major MeSH] OR "anger control"[Title/Abstract] OR “anger management”[Title/Abstract] OR "attention deficit*"[Title/Abstract] OR “Attention Deficit and Disruptive Behavior Disorders”[Major MeSH] OR "attention problem*"[Title/Abstract] OR "behavior problem*"[Title/Abstract] OR "behaviour problem*"[Title/Abstract] OR “Problem Behavior”[Major MeSH] OR "behavioral development*"[Title/Abstract] OR "behavioural development*"[Title/Abstract] OR "behavioral outcome*"[Title/Abstract] OR "behavioural outcome*"[Title/Abstract] OR "behavioral well-being"[Title/Abstract] OR "behavioural well-being"[Title/Abstract] OR "behavioral wellbeing"[Title/Abstract] OR "behavioural wellbeing"[Title/Abstract] OR "disruptive behavior*"[Title/Abstract] OR "disruptive behaviour*"[Title/Abstract] OR "intermittent explosive disorder*"[Title/Abstract] OR “impulse control disorder*”[Title/Abstract] OR “Disruptive, Impulse Control, and Conduct Disorders”[Major MeSH] OR "oppositional defiant disorder*"[Title/Abstract] OR aggress*[Title/Abstract] OR Aggression[Major MeSH] OR antagonistic[Title/Abstract] OR antisocial[Title/Abstract] OR “anti-social”[Title/Abstract] OR “anti social”[Title/Abstract] OR “Antisocial Personality Disorder”[Major MeSH] OR “conduct disorder*”[Title/Abstract] OR “Conduct Disorder”[Major MeSH] OR disinhibit*[Title/Abstract] OR externaliz*[Title/Abstract] OR hyperactiv*[Title/Abstract] OR hyperkine*[Title/Abstract] OR impulsiv*[Title/Abstract] OR “Impulsive Behavior”[Major MeSH] OR inattenti*[Title/Abstract] OR irritab*[Title/Abstract] OR sociopath*[Title/Abstract] OR “substance use*”[Title/Abstract] OR “behaviostance abuse*”[Title/Abstract] OR addict*[Title/Abstract] OR “drug dependenc*”[Title/Abstract] OR “Substance-Related Disorders”[Major MeSH] OR "communication disorder*"[Title/Abstract] OR “Communication Disorders”[Major MeSH] OR "coordination disorder*"[Title/Abstract] OR "developmental delay*"[Title/Abstract] OR "developmental disorder*"[Title/Abstract] OR “development disorder*”[Title/Abstract] OR “developmental disabilit*”[Title/Abstract] OR “Developmental Disabilities”[Major MeSH] OR "fluency disorder*"[Title/Abstract] OR "intellectual disab*"[Title/Abstract] OR “intellectual impairment*”[Title/Abstract] OR “Intellectual Disability”[Major MeSH] OR "language disorder*"[Title/Abstract] OR “Language Disorders”[Major MeSH] OR “Language Development Disorders”[Major MeSH] OR “language development disorder*”[Title/Abstract] OR “language developmental disorder*”[Title/Abstract] OR “developmental language disorder*”[Title/Abstract] OR “language disabilit*”[Title/Abstract] OR "learning disorder*"[Title/Abstract] OR “learning disabilit*”[Title/Abstract] OR “Learning Disabilities”[Major MeSH] OR "motor disorder*"[Title/Abstract] OR “motor dysfunction*”[Title/Abstract] OR “Motor Disorders”[Major MeSH] OR "movement disorder*"[Title/Abstract] OR “Movement Disorders”[Major MeSH] OR "speech sound disorder*"[Title/Abstract] OR “Speech Sound Disorder”[Major MeSH] OR “Speech Disorders”[Major MeSH] OR “speech disorder*”[Title/Abstract] OR “articulation disorder*”[Title/Abstract] OR autis*[Title/Abstract] OR “Autism Spectrum Disorder”[Major MeSH] OR “Autistic Disorder”[Major MeSH] OR “Asperger Syndrome”[Major MeSH] OR asperger*[Title/Abstract] OR “neurodevelopmental disorder*”[Title/Abstract] OR “neuro-developmental disorder*”[Title/Abstract] OR “Neurodevelopmental Disorders”[Major MeSH] OR stutter*[Title/Abstract] OR Stuttering[Major MeSH] OR tic[Title/Abstract] OR tics[Title/Abstract] OR Tics[Major MeSH] OR Tourette*[Title/Abstract] OR “Tourette Syndrome”[Major MeSH] OR "posttraumatic stress disorder*"[Title/Abstract] OR “post-traumatic stress disorder*”[Title/Abstract] OR “post traumatic stress disorder*”[Title/Abstract] OR “Stress Disorders, Post-Traumatic”[Major MeSH] OR "mental disorder*"[Title/Abstract] OR “Mental Disorders”[majr:noexp] OR "mental illness*"[Title/Abstract] OR "psychological distress*"[Title/Abstract] OR “Psychological Distress”[Major MeSH] OR "psychological impair*"[Title/Abstract] OR “distress syndrome*”[Title/Abstract] OR "psychological symptom*"[Title/Abstract] OR psychopatho*[Title/Abstract] OR Psychopathology[Major MeSH]) |
| #4 | Population Group | (adolescen*[Title/Abstract] OR child[Title/Abstract] OR children[Title/Abstract] OR teen*[Title/Abstract] OR boys[Title/Abstract] OR boy[Title/Abstract] OR girls[Title/Abstract] OR girl[Title/Abstract] OR youth[Title/Abstract] OR youths[Title/Abstract] OR "Adolescent"[Mesh] OR "Child"[Mesh:Noexp]) |
| #5 |  | #1 AND #2 AND #3 AND #4 |
| #6 | Limits applied: Language | #5 AND English[lang] |
|  | Limits applied: Exclude specific publication type | #6 NOT (letter[Publication Type] OR editorial[Publication Type] OR comment[Publication Type] OR news[Publication Type] OR editorial[Title/Abstract] OR commentary[Title/Abstract] OR "Published Erratum"[Publication Type] OR errata[Title/Abstract] OR erratum[Title/Abstract] OR corrigenda[Title/Abstract] OR corrigendum[Title/Abstract] OR protocol[Title/Abstract] OR protocols[Title/Abstract] OR “meta-analysis”[Title/Abstract] OR “meta-analyses”[Title/Abstract] OR metanalyses[Title/Abstract] OR metanalysis[Title/Abstract] OR metaanalyses[Title/Abstract] OR metaanalysis[Title/Abstract] OR “meta analyses”[Title/Abstract] OR “meta analysis”[Title/Abstract] OR "Review"[Publication Type] OR “systematic review*”[Title/Abstract] OR "Systematic Review"[Publication Type] OR "Meta-Analysis" [Publication Type] OR "Network Meta-Analysis"[Mesh] OR "Review"[Publication Type] OR “integrative review”[Title/Abstract] OR "Case Reports" [Publication Type] OR "case report*"[Title/Abstract] OR "case series"[Title/Abstract] OR "case presentation*"[Title/Abstract] OR "Single-Case Studies as Topic"[Mesh] OR "case stud*"[Title/Abstract] OR "case histor*"[Title/Abstract] OR "single case design*"[Title/Abstract] OR "single-case*"[Title/Abstract] OR "single-case analysis"[Title/Abstract] OR "single cases"[Title/Abstract]) |

**Notes:** The limit for language (English) was applied to the main search using the filters available in PubMed. The keywords were searched in the title and abstract fields in PubMed (i.e., [Title/Abstract]), text field (i.e., [Text Word]), and the controlled vocabulary terms are indicated with [Mesh] or [Major MeSH] where the MeSH terms is the main focus of the article. Terms searched in the [Text Word] field are searched in the title, abstract, keywords, and MeSH fields. Phrases were enclosed in quotation marks to force the searching of the exact terms in order presented. To these results, the search strategy to exclude specific publication types specified in the eligibility criteria was used. No other limits were applied to the searches.

**Database:** Web of Science: Core Collection*
**Platform:** Clarivate Analytics
**Date Searched:** January 10, 2023

**Database Date Coverage:** 1900–present
**Date Limits:** None
**Other Limits/Filters:** Language: English; Document types: primary research only

| **Set** | **Concept** | **Search Strategy** |
| --- | --- | --- |
| #1 |  | TS=((("ecological momentary assessment*" OR "ecological momentary intervention*" OR momentary OR "daily diary" OR "daily diaries" OR "electronic diar*" OR "e diar*" OR "e-diar*" OR ediar* OR "digital diar*" OR "event sampling*" OR "experience sampling*" OR "experience-sampling*" OR "real time assessment*" OR "real-time assessment*" OR "just-in-time assessment*" OR "just in time assessment*" OR "short message service*" OR "ambulatory assessment*" OR "text messag*" OR texting OR "daily prompt*" OR "electronic prompt*" OR "digital prompt*") NOT sexting)) |
| #2 |  | TS=(cellphone* OR "cell phone*" OR "cellular phone*" OR "cellular telephone*" OR "mobile telephone*" OR "mobile phone*" OR smartphone* OR "palm pilot*" OR "mobile device*" OR "mobile technolog*" OR app OR apps OR "mobile application*" OR computer OR computers OR tablet* OR iPad* OR laptop* OR iphone* OR iPhone* OR Android) |
| #3 |  | TS=(somatoform OR "eating disorder*" OR "eating patho*" OR "obsessive compulsive disorder*" OR "personality disorder*" OR "self harm*" OR "self-harm*" OR "self-injur*" OR "self injur*" OR automutilat* OR "self mutilate*" OR "self-multilat*" OR "self inflicted injur*" OR "sexual problem*" OR "gender dysphori*" OR "gender identity disorder*" OR "psychosexual disorder*" OR anorex* OR anxiety OR anxieties OR anxious* OR "binge eat*" OR "binge-eat*" OR bipolar OR bulim* OR depressi* OR dysthymi* OR fear* OR hypomani* OR internaliz* OR mania* OR manic OR panic OR panics OR panicked OR phobi* OR suicid* OR trauma* OR "thought disorder*" OR paranoi* OR psychosis OR psychotic OR schizo* OR "anger control" OR "anger management" OR "attention deficit*" OR "attention problem*" OR "behavior problem*" OR "behaviour problem*" OR "behavioral development*" OR "behavioural development*" OR "behavioral outcome*" OR "behavioural outcome*" OR "behavioral well-being" OR "behavioural well-being" OR "behavioral wellbeing" OR "behavioural wellbeing" OR "disruptive behavior*" OR "disruptive behaviour*" OR "intermittent explosive disorder*" OR "impulse control disorder*" OR "oppositional defiant disorder*" OR aggress* OR antagonistic OR antisocial OR "anti-social" OR "anti social" OR "conduct disorder*" OR disinhibit* OR externaliz* OR hyperactiv* OR hyperkine* OR impulsiv* OR inattenti* OR irritab* OR sociopath* OR "substance use*" OR "substance abuse*" OR addict* OR "drug dependenc*" OR "communication disorder*" OR "coordination disorder*" OR "developmental delay*" OR "developmental disorder*" OR "development disorder*" OR "developmental disabilit*" OR "fluency disorder*" OR "intellectual disab*" OR "intellectual impairment*" OR "language disorder*" OR "language development disorder*" OR "language developmental disorder*" OR "developmental language disorder*" OR "language disabilit*" OR "learning disorder*" OR "learning disabilit*" OR "motor disorder*" OR "motor dysfunction*" OR "movement disorder*" OR "speech sound disorder*" OR "speech disorder*" OR "articulation disorder*" OR autis* OR asperger* OR "neurodevelopmental disorder*" OR "neuro-developmental disorder*" OR stutter* OR tic OR tics OR Tourette* OR "posttraumatic stress disorder*" OR "post-traumatic stress disorder*" OR "post traumatic stress disorder*" OR "mental disorder*" OR "mental illness*" OR "psychological distress*" OR "psychological impair*" OR "distress syndrome*" OR "psychological symptom*" OR psychopatho*) |
| #4 |  | TS=(adolescen* OR child OR children OR teen* OR boys OR boy OR girls OR girl OR youth OR youths) |
| #5 |  | #1 AND #2 AND #3 AND #4 |
| #6 | Limit applied: Language | #5 AND Language: English |
| #7 | Limit applied: Document types | #6 AND Document Types: Article OR Early Access OR Correction |
| #8 | Limit applied: Exclude specific publication types | #7 NOT TI=(editorial OR commentary OR "conference abstract*" OR "conference proceeding*" OR symposium* OR errata OR erratum OR corrigenda OR corrigendum OR protocol OR protocols OR "meta-analysis" OR "meta-analyses" OR metanalyses OR metanalysis OR metaanalyses OR metaanalysis OR "meta analyses" OR "meta analysis" OR "systematic review*" OR "integrative review") NOT DT=(Editorial Material OR Letter OR News Item OR Note OR Book OR Book Chapter OR Excerpt OR Item About an Individual OR Meeting Abstract OR Meeting Summary OR Reprint OR Review OR Retracted Publication OR Retraction) |

**Notes:** The limits for language (English) and Document Type (Article, Early Access, Correction) were applied to the main search using the filters available. The keywords were searched in the Topic field (i.e., TS) which searches the title, abstract, author keywords, and KeyWords Plus fields. Phrases were enclosed in quotation marks to force the searching of the exact terms in order presented. A search strategy was used to exclude specific publication types as specified in the eligibility criteria. No other limits were applied to the searches.

*Science Citation Index Expanded (SCI-EXPANDED)--1900-present

Social Sciences Citation Index (SSCI)--1900-present

Conference Proceedings Citation Index – Science (CPCI-S)--1990-present

Conference Proceedings Citation Index – Social Science & Humanities (CPCI-SSH)--1990-present

Book Citation Index – Science (BKCI-S)--2005-present

Book Citation Index – Social Sciences & Humanities (BKCI-SSH)--2005-present

Emerging Sources Citation Index (ESCI)--2005-present

Current Chemical Reactions (CCR-EXPANDED)--1985-present

Index Chemicus (IC)--1993-present

**Database:** PsycNet: PsycInfo & PsycArticles
**Platform:** American Psychological Association
**Date Searched:** January 11, 2023

**Database Date Coverage:** 1800–present
**Date Limits:** None
**Other Limits/Filters:** Publication type: Peer Reviewed Journal; Notes: Use Advanced Search. Search keywords in the title and abstract fields. Search controlled vocabulary terms in the Index Terms and MeSH fields. Run main search first and to these results exclude all other publication types and limiting to Peer Reviewed Journal.

| **Set** | **Concept** | **Search Strategy** |
| --- | --- | --- |
| #1 | Ecological Momentary Assessment | Title: (("ecological momentary assessment*" OR "ecological momentary intervention*" OR momentary OR "daily diary" OR "daily diaries" OR "electronic diar*" OR "e diar*" OR "e-diar*" OR ediar* OR "digital diar*" OR "event sampling*" OR "experience sampling*" OR "experience-sampling*" OR "real time assessment*" OR "real-time assessment*" OR "just-in-time assessment*" OR "just in time assessment*" OR "short message service*" OR "ambulatory assessment*" OR "text messag*" OR texting OR "daily prompt*" OR "electronic prompt*" OR "digital prompt*") |
| #2 | Ecological Momentary Assessment | Abstract: (("ecological momentary assessment*" OR "ecological momentary intervention*" OR momentary OR "daily diary" OR "daily diaries" OR "electronic diar*" OR "e diar*" OR "e-diar*" OR ediar* OR "digital diar*" OR "event sampling*" OR "experience sampling*" OR "experience-sampling*" OR "real time assessment*" OR "real-time assessment*" OR "just-in-time assessment*" OR "just in time assessment*" OR "short message service*" OR "ambulatory assessment*" OR "text messag*" OR texting OR "daily prompt*" OR "electronic prompt*" OR "digital prompt*") |
| #3 | Ecological Momentary Assessment | Index Terms: ({Ecological Momentary Assessment} OR {Journal Writing} OR {Text Messaging}) |
| #4 | Ecological Momentary Assessment | MeSH Terms: ("Ecological Momentary Assessment" OR "Diaries as Topic" OR "Text Messaging") |
| #5 | Ecological Momentary Assessment | #1 OR #2 OR #3 OR #4 |
| #6 | Digital/Mobile Devices | Title: (cellphone* OR "cell phone*" OR "cellular phone*" OR "cellular telephone*" OR "mobile telephone*" OR "mobile phone*" OR smartphone* OR "palm pilot*" OR "mobile device*" OR "mobile technolog*" OR app OR apps OR "mobile application*" OR computer OR computers OR tablet* OR iPad* OR laptop* OR iphone* OR iPhone* OR Android) |
| #7 | Digital/Mobile Devices | Abstract: (cellphone* OR "cell phone*" OR "cellular phone*" OR "cellular telephone*" OR "mobile telephone*" OR "mobile phone*" OR smartphone* OR "palm pilot*" OR "mobile device*" OR "mobile technolog*" OR app OR apps OR "mobile application*" OR computer OR computers OR tablet* OR iPad* OR laptop* OR iphone* OR iPhone* OR Android) |
| #8 | Digital/Mobile Devices | Index Terms: ({Mobile Devices} OR {Mobile Phones} OR {Smartphones} OR {Tablet Computers} OR {Computer Applications} OR {Mobile Applications}) |
| #9 | Digital/Mobile Devices | MeSH Terms: ("Smartphone" OR "Cell Phone" OR "Mobile Applications" OR "Computers, Handheld") |
| #10 | Digital/Mobile Devices | #6 OR #7 OR #8 OR #9 |
| #11 | Psychological Symptoms/Psychopathologies | Title: (somatoform OR "eating disorder*" OR "eating patho*" OR "obsessive compulsive disorder*" OR "personality disorder*" OR "self harm*" OR "self-harm*" OR "self-injur*" OR "self injur*" OR automutilat* OR "self mutilate*" OR "self-multilat*" OR "self inflicted injur*" OR "sexual problem*" OR "gender dysphori*" OR "gender identity disorder*" OR "psychosexual disorder*" OR anorex* OR anxiety OR anxieties OR anxious* OR "binge eat*" OR "binge-eat*" OR bipolar OR bulim* OR depressi* OR dysthymi* OR fear* OR hypomani* OR internaliz* OR mania* OR manic OR panic OR panics OR panicked OR phobi* OR suicid* OR trauma* OR "thought disorder*" OR paranoi* OR psychosis OR psychotic OR schizo* OR "anger control" OR "anger management" OR "attention deficit*" OR "attention problem*" OR "behavior problem*" OR "behaviour problem*" OR "behavioral development*" OR "behavioural development*" OR "behavioral outcome*" OR "behavioural outcome*" OR "behavioral well-being" OR "behavioural well-being" OR "behavioral wellbeing" OR "behavioural wellbeing" OR "disruptive behavior*" OR "disruptive behaviour*" OR "intermittent explosive disorder*" OR "impulse control disorder*" OR "oppositional defiant disorder*" OR aggress* OR antagonistic OR antisocial OR "anti-social" OR "anti social" OR "conduct disorder*" OR disinhibit* OR externaliz* OR hyperactiv* OR hyperkine* OR impulsiv* OR inattenti* OR irritab* OR sociopath* OR "substance use*" OR "substance abuse*" OR addict* OR "drug dependenc*" OR "communication disorder*" OR "coordination disorder*" OR "developmental delay*" OR "developmental disorder*" OR "development disorder*" OR "developmental disabilit*" OR "fluency disorder*" OR "intellectual disab*" OR "intellectual impairment*" OR "language disorder*" OR "language development disorder*" OR "language developmental disorder*" OR "developmental language disorder*" OR "language disabilit*" OR "learning disorder*" OR "learning disabilit*" OR "motor disorder*" OR "motor dysfunction*" OR "movement disorder*" OR "speech sound disorder*" OR "speech disorder*" OR "articulation disorder*" OR autis* OR asperger* OR "neurodevelopmental disorder*" OR "neuro-developmental disorder*" OR stutter* OR tic OR tics OR Tourette* OR "posttraumatic stress disorder*" OR "post-traumatic stress disorder*" OR "post traumatic stress disorder*" OR "mental disorder*" OR "mental illness*" OR "psychological distress*" OR "psychological impair*" OR "distress syndrome*" OR "psychological symptom*" OR psychopatho*) |
| #12 | Psychological Symptoms/Psychopathologies | Abstract: (somatoform OR "eating disorder*" OR "eating patho*" OR "obsessive compulsive disorder*" OR "personality disorder*" OR "self harm*" OR "self-harm*" OR "self-injur*" OR "self injur*" OR automutilat* OR "self mutilate*" OR "self-multilat*" OR "self inflicted injur*" OR "sexual problem*" OR "gender dysphori*" OR "gender identity disorder*" OR "psychosexual disorder*" OR anorex* OR anxiety OR anxieties OR anxious* OR "binge eat*" OR "binge-eat*" OR bipolar OR bulim* OR depressi* OR dysthymi* OR fear* OR hypomani* OR internaliz* OR mania* OR manic OR panic OR panics OR panicked OR phobi* OR suicid* OR trauma* OR "thought disorder*" OR paranoi* OR psychosis OR psychotic OR schizo* OR "anger control" OR "anger management" OR "attention deficit*" OR "attention problem*" OR "behavior problem*" OR "behaviour problem*" OR "behavioral development*" OR "behavioural development*" OR "behavioral outcome*" OR "behavioural outcome*" OR "behavioral well-being" OR "behavioural well-being" OR "behavioral wellbeing" OR "behavioural wellbeing" OR "disruptive behavior*" OR "disruptive behaviour*" OR "intermittent explosive disorder*" OR "impulse control disorder*" OR "oppositional defiant disorder*" OR aggress* OR antagonistic OR antisocial OR "anti-social" OR "anti social" OR "conduct disorder*" OR disinhibit* OR externaliz* OR hyperactiv* OR hyperkine* OR impulsiv* OR inattenti* OR irritab* OR sociopath* OR "substance use*" OR "substance abuse*" OR addict* OR "drug dependenc*" OR "communication disorder*" OR "coordination disorder*" OR "developmental delay*" OR "developmental disorder*" OR "development disorder*" OR "developmental disabilit*" OR "fluency disorder*" OR "intellectual disab*" OR "intellectual impairment*" OR "language disorder*" OR "language development disorder*" OR "language developmental disorder*" OR "developmental language disorder*" OR "language disabilit*" OR "learning disorder*" OR "learning disabilit*" OR "motor disorder*" OR "motor dysfunction*" OR "movement disorder*" OR "speech sound disorder*" OR "speech disorder*" OR "articulation disorder*" OR autis* OR asperger* OR "neurodevelopmental disorder*" OR "neuro-developmental disorder*" OR stutter* OR tic OR tics OR Tourette* OR "posttraumatic stress disorder*" OR "post-traumatic stress disorder*" OR "post traumatic stress disorder*" OR "mental disorder*" OR "mental illness*" OR "psychological distress*" OR "psychological impair*" OR "distress syndrome*" OR "psychological symptom*" OR psychopatho*) |
| #13 | Psychological Symptoms/Psychopathologies | Index Terms: ({Somatoform Disorders} OR {Anorexia Nervosa} OR {Bulimia} OR {Sexual Function Disturbances} OR {Obsessive Compulsive Disorder} OR {Anxiety Disorders} OR {Personality Disorders} OR {Antisocial Personality Disorder} OR {Avoidant Personality Disorder} OR {Borderline Personality Disorder} OR {Dependent Personality Disorder} OR {Histrionic Personality Disorder} OR {Narcissistic Personality Disorder} OR {Obsessive Compulsive Personality Disorder} OR {Paranoid Personality Disorder} OR {Passive Aggressive Personality Disorder} OR {Sadomasochistic Personality} OR {Schizoid Personality Disorder} OR {Schizotypal Personality Disorder} OR {Body Dysmorphic Disorder} OR {Conversion Disorder} OR {Factitious Disorders} OR {Illness Anxiety Disorder} OR {Neurasthenia} OR {Somatization Disorder} OR {Somatoform Pain Disorder} OR {Eating Disorders} OR {Binge Eating Disorder} OR {Feeding Disorders} OR {Hyperphagia} OR {Kleine Levin Syndrome} OR {Orthorexia} OR {Pica} OR {Purging (Eating Disorders)} OR {Rumination (Eating)} OR {Hoarding Disorder} OR {Koro} OR {Dyspareunia} OR {Erectile Dysfunction} OR {Female Sexual Dysfunction} OR {Inhibited Sexual Desire} OR {Premature Ejaculation} OR {Priapism} OR {Vaginismus} OR {Gender Dysphoria} OR {Anxiety} OR {Anxiety Sensitivity} OR {Climate Anxiety} OR {Computer Anxiety} OR {Death Anxiety} OR {Health Anxiety} OR {Mathematics Anxiety} OR {Performance Anxiety} OR {Social Anxiety} OR {Speech Anxiety} OR {Test Anxiety} OR {Travel Anxiety} OR {Castration Anxiety} OR {Generalized Anxiety Disorder} OR {Phobias} OR {Separation Anxiety Disorder} OR {Trichotillomania} OR {Bipolar Disorder} OR {Bipolar I Disorder} OR {Bipolar II Disorder} OR {Cyclothymic Disorder} OR {Mania} OR {Depression (Emotion)} OR {Major Depression} OR {Anaclitic Depression} OR {Dysthymic Disorder} OR {Endogenous Depression} OR {Late Life Depression} OR {Postpartum Depression} OR {Reactive Depression} OR {Recurrent Depression} OR {Treatment Resistant Depression} OR {Fear} OR {Panic} OR {Panic Attack} OR {Panic Disorder} OR {Suicide} OR {Youth Suicide} OR {Nonsuicidal Self-Injury} OR {Head Banging} OR {Self-Inflicted Wounds} OR {Self-Poisoning} OR {Paranoid Psychosis} OR {Psychosis} OR {Affective Psychosis} OR {Alcohol Induced Psychotic Disorders} OR {Brief Psychotic Disorder} OR {Capgras Syndrome} OR {Childhood Onset Psychosis} OR {Chronic Psychosis} OR {Experimental Psychosis} OR {Hallucinosis} OR {Postpartum Psychosis} OR {Reactive Psychosis} OR {Schizophrenia} OR {Senile Dementia} OR {Substance Induced Psychotic Disorders} OR {Acute Schizophrenia} OR {Catatonic Schizophrenia} OR {Childhood Onset Schizophrenia} OR {Paranoid Schizophrenia} OR {Process Schizophrenia} OR {Schizoaffective Disorder} OR {Schizophrenia (Disorganized Type)} OR {Schizophreniform Disorder} OR {Undifferentiated Schizophrenia} OR {Attention Deficit Disorder} OR {Attention Deficit Disorder With Hyperactivity} OR {Oppositional Defiant Disorder} OR {Disruptive Behavior Disorders} OR {Conduct Disorder} OR {Behavior Disorders} OR {Impulse Control Disorders} OR {Kleptomania} OR {Pyromania} OR {Self-Destructive Behavior} OR {Aggressive Behavior} OR {Explosive Disorder} OR {Antisocial Behavior} OR {Impulsiveness} OR {Aggressive Driving Behavior} OR {Attack Behavior} OR {Coercion} OR {Conflict} OR {Microaggression} OR {Relational Aggression} OR {Threat} OR {Antisocial Behavior} OR {Conduct Disorder} OR {Drug Abuse} OR {Drug Addiction} OR {Communication Disorders} OR {Hearing Disorders} OR {Language Disorders} OR {Agnosia} OR {Agraphia} OR {Dyscalculia} OR {Dyslexia} OR {Echolalia} OR {Mutism} OR {Specific Language Impairment} OR {Speech Disorders} OR {Developmental Disabilities} OR {Intellectual Development Disorder} OR {Anencephaly} OR {Crying Cat Syndrome} OR {Down's Syndrome} OR {Tay Sachs Disease} OR {Williams Syndrome} OR {Prader Willi Syndrome} OR {Fragile X Syndrome} OR {Rett Syndrome} OR {Learning Disabilities} OR {Movement Disorders} OR {Alien Limb Syndrome} OR {Apraxia} OR {Ataxia} OR {Athetosis} OR {Catalepsy} OR {Cataplexy} OR {Chorea} OR {Dyskinesia} OR {Dyspraxia} OR {Myasthenia Gravis} OR {Paralysis} OR {Spasms} OR {Tics} OR {Torticollis} OR {Tremor} OR {Aphasia} OR {Articulation Disorders} OR {Dysphonia} OR {Stuttering} OR {Autism Spectrum Disorders} OR {Autistic Traits} OR {Neurodevelopmental Disorders} OR {Autism Spectrum Disorders} OR {Developmental Disabilities} OR {Emotional and Behavioral Disorders} OR {Learning Disorders} OR {Stuttering} OR {Posttraumatic Stress Disorder} OR {Complex PTSD} OR {DESNOS} OR {Psychological Stress} OR {Psychopathology} OR {Adolescent Psychopathology} OR {Child Psychopathology} OR {Mental Disorders} OR {Internalizing Symptoms} OR {Internalization} OR {Externalization} OR {Externalizing Symptoms}) |
| #14 | Psychological Symptoms/Psychopathologies | MeSH Terms: ("Somatoform Disorders" OR "Feeding and Eating Disorders" OR "Obsessive-Compulsive Disorder" OR "Personality Disorders" OR "Self-Injurious Behavior" OR "Sexual Dysfunctions, Psychological" OR "Sexual and Gender Disorders" OR Anorexia OR "Anorexia Nervosa" OR Anxiety OR "Anxiety Disorders" OR "Binge-Eating Disorder" OR "Bipolar Disorder" OR Bulimia OR Depression OR "Depressive Disorder" OR "Dysthymic Disorder" OR Fear OR Mania OR Panic OR "Panic Disorder" OR "Phobic Disorders" OR Suicide OR "Paranoid Disorders" OR "Psychotic Disorders" OR Schizophrenia OR "Attention Deficit and Disruptive Behavior Disorders" OR "Problem Behavior" OR "Disruptive, Impulse Control, and Conduct Disorders" OR Aggression OR "Antisocial Personality Disorder" OR "Conduct Disorder" OR "Impulsive Behavior" OR "Substance-Related Disorders" OR "Communication Disorders" OR "Developmental Disabilities" OR "Intellectual Disability" OR "Language Disorders" OR "Language Development Disorders" OR "Learning Disabilities" OR "Motor Disorders" OR "Movement Disorders" OR "Speech Sound Disorder" OR "Speech Disorders" OR "Autism Spectrum Disorder" OR "Autistic Disorder" OR "Asperger Syndrome" OR "Neurodevelopmental Disorders" OR Stuttering OR Tics OR "Tourette Syndrome" OR "Stress Disorders, Post-Traumatic" OR "Mental Disorders" OR "Psychological Distress" OR Psychopathology) |
| #15 | Psychological Symptoms/Psychopathologies | #11 OR #12 OR #13 OR #14 |
| #16 | Population Group | Title: (adolescen* OR child OR children OR teen* OR boys OR boy OR girls OR girl OR youth OR youths) |
| #17 | Population Group | Abstract: (adolescen* OR child OR children OR teen* OR boys OR boy OR girls OR girl OR youth OR youths) |
| #18 | Population Group | MeSH Terms: ("Adolescent" OR "Child") |
| #19 | Population Group | #16 OR #17 OR #18 |
| #20 | Combined | #5 AND #10 AND #15 |
| #21 | Combined | #20 AND #19 |
| #22 | Excluding irrelevant concept | #21 NOT ((Title:(sexting)) OR (Abstract:(sexting)) OR IndexTermsFilt:("Sexting"))) |
| #23 | Limit applied: Specific publication type | #22 AND Publication Type: Peer Reviewed Journal |

**Notes:** The limit for Publication Type was applied to the main search using the filter available. The keywords were searched in the title, abstract fields. The controlled vocabulary terms used were searched in the Index Terms and MeSH Terms fields and indicated by curly brackets (i.e., { }). Phrases were enclosed in quotation marks to force the searching of the exact terms in order presented. No other limits were applied to the searches.

**Database:** Scopus
**Platform:** Elsevier
**Date Searched:** January 10, 2023

**Database Date Coverage:** 1788–present

**Date Limits:** None
**Other Limits/Filters:** Language: English; Document type: primary research only

| **Set** | **Concept** | **Search Strategy** |
| --- | --- | --- |
| #1 |  | Title-Abs-Key( (("ecological momentary assessment*" OR "ecological momentary intervention*" OR momentary OR "daily diary" OR "daily diaries" OR "electronic diar*" OR "e diar*" OR "e-diar*" OR ediar* OR "digital diar*" OR "event sampling*" OR "experience sampling*" OR "experience-sampling*" OR "real time assessment*" OR "real-time assessment*" OR "just-in-time assessment*" OR "just in time assessment*" OR "short message service*" OR "ambulatory assessment*" OR "text messag*" OR texting OR "daily prompt*" OR "electronic prompt*" OR "digital prompt*") AND NOT sexting)) |
| #2 |  | Title-Abs-Key(cellphone* OR "cell phone*" OR "cellular phone*" OR "cellular telephone*" OR "mobile telephone*" OR "mobile phone*" OR smartphone* OR "palm pilot*" OR "mobile device*" OR "mobile technolog*" OR app OR apps OR "mobile application*" OR computer OR computers OR tablet* OR iPad* OR laptop* OR iphone* OR iPhone* OR Android) |
| #3 |  | Title-Abs-Key(somatoform OR "eating disorder*" OR "eating patho*" OR "obsessive compulsive disorder*" OR "personality disorder*" OR "self harm*" OR "self-harm*" OR "self-injur*" OR "self injur*" OR automutilat* OR "self mutilate*" OR "self-multilat*" OR "self inflicted injur*" OR "sexual problem*" OR "gender dysphori*" OR "gender identity disorder*" OR "psychosexual disorder*" OR anorex* OR anxiety OR anxieties OR anxious* OR "binge eat*" OR "binge-eat*" OR bipolar OR bulim* OR depressi* OR dysthymi* OR fear* OR hypomani* OR internaliz* OR mania* OR manic OR panic OR panics OR panicked OR phobi* OR suicid* OR trauma* OR "thought disorder*" OR paranoi* OR psychosis OR psychotic OR schizo* OR "anger control" OR "anger management" OR "attention deficit*" OR "attention problem*" OR "behavior problem*" OR "behaviour problem*" OR "behavioral development*" OR "behavioural development*" OR "behavioral outcome*" OR "behavioural outcome*" OR "behavioral well-being" OR "behavioural well-being" OR "behavioral wellbeing" OR "behavioural wellbeing" OR "disruptive behavior*" OR "disruptive behaviour*" OR "intermittent explosive disorder*" OR "impulse control disorder*" OR "oppositional defiant disorder*" OR aggress* OR antagonistic OR antisocial OR "anti-social" OR "anti social" OR "conduct disorder*" OR disinhibit* OR externaliz* OR hyperactiv* OR hyperkine* OR impulsiv* OR inattenti* OR irritab* OR sociopath* OR "substance use*" OR "substance abuse*" OR addict* OR "drug dependenc*" OR "communication disorder*" OR "coordination disorder*" OR "developmental delay*" OR "developmental disorder*" OR "development disorder*" OR "developmental disabilit*" OR "fluency disorder*" OR "intellectual disab*" OR "intellectual impairment*" OR "language disorder*" OR "language development disorder*" OR "language developmental disorder*" OR "developmental language disorder*" OR "language disabilit*" OR "learning disorder*" OR "learning disabilit*" OR "motor disorder*" OR "motor dysfunction*" OR "movement disorder*" OR "speech sound disorder*" OR "speech disorder*" OR "articulation disorder*" OR autis* OR asperger* OR "neurodevelopmental disorder*" OR "neuro-developmental disorder*" OR stutter* OR tic OR tics OR Tourette* OR "posttraumatic stress disorder*" OR "post-traumatic stress disorder*" OR "post traumatic stress disorder*" OR "mental disorder*" OR "mental illness*" OR "psychological distress*" OR "psychological impair*" OR "distress syndrome*" OR "psychological symptom*" OR psychopatho*) |
| #4 |  | Title-Abs-Key(adolescen* OR child OR children OR teen* OR boys OR boy OR girls OR girl OR youth OR youths) |
| #5 |  | #1 AND #2 AND #3 AND #4 |
| #6 | Limit applied: Language | #5 AND ( LIMIT-TO ( LANGUAGE , "English" ) ) |
| #7 | Limit applied: Exclude specific publication type | #6 AND NOT DOCTYPE ( ab OR bk OR ch OR cp OR cr OR bz OR dp OR ed OR er OR le OR mm OR no OR pr OR rp OR re OR sh ) AND NOT TITLE ( editorial OR commentary OR {conference abstract*} OR {conference proceeding*} OR symposium* OR errata OR erratum OR corrigenda OR corrigendum OR protocol OR protocols OR {meta-analysis} OR {meta-analyses} OR metanalyses OR metanalysis OR metaanalyses OR metaanalysis OR {meta analyses} OR {meta analysis} OR {systematic review*} OR {integrative review} ) |

**Notes:** The limits language (English) and Document Type were applied to the main search using the filters available. The keywords were searched in the title, abstract, or keywords fields. [Phrases](https://service.elsevier.com/app/answers/detail/a_id/11365/supporthub/scopus/kw/phrase+searching/) were enclosed in curly brackets (i.e., { }) to force the searching of the exact terms in order presented, or double quotation marks (i.e., “x”) to find approximate phrases. The search terms to limit by publication type was searched in the Title field only (i.e., TITLE) and the Document Type field (i.e., DOCTYPE). A search strategy was used to exclude specific publication types as specified in the eligibility criteria. No other limits were applied to the searches.

**Database:** Embase
**Platform:** Elsevier
**Date Searched:** January 10, 2023

**Database Date Coverage:** 1947–present
**Date Limits:** None
**Other Limits/Filters:** Language: English; Source: Embase and Embase Classic; Publication type: Primary research only (excluding specific publication types specified in the eligibility criteria). Uncheck all Mapping checkboxes on the main search page

| **Set** | **Concept** | **Search Strategy** |
| --- | --- | --- |
| #1 | Ecological Momentary Assessment | (('ecological momentary assessment'/exp OR 'text messaging'/exp OR 'ecological momentary assessment*':ti,ab OR 'ecological momentary intervention*':ti,ab OR momentary:ti,ab OR 'daily diary':ti,ab OR 'daily diaries':ti,ab OR 'electronic diar*':ti,ab OR 'e diar*':ti,ab OR 'e-diar*':ti,ab OR ediar*:ti,ab OR 'digital diar*':ti,ab OR 'event sampling*':ti,ab OR 'experience sampling*':ti,ab OR 'experience-sampling*':ti,ab OR 'just-in-time assessment*':ti,ab OR 'just in time assessment*':ti,ab OR 'real time assessment*':ti,ab OR 'real-time assessment*':ti,ab OR 'short message service*':ti,ab OR 'ambulatory assessment*':ti,ab OR 'text messag*':ti,ab OR texting:ti,ab OR 'daily prompt*':ti,ab OR 'electronic prompt*':ti,ab OR 'digital prompt*':ti,ab) NOT sexting:ti,ab) |
| #2 | Digital/Mobile Devices | ('smartphone'/exp OR 'mobile phone'/exp OR 'mobile application'/exp OR 'personal digital assistant'/exp OR 'tablet computer'/exp OR cellphone*:ti,ab OR 'cell phone*':ti,ab OR 'cellular phone*':ti,ab OR 'cellular telephone*':ti,ab OR 'mobile telephone*':ti,ab OR 'mobile phone*':ti,ab OR smartphone*:ti,ab OR 'palm pilot*':ti,ab OR 'mobile device*':ti,ab OR 'mobile technolog*':ti,ab OR app:ti,ab OR apps:ti,ab OR 'mobile application*':ti,ab OR computer:ti,ab OR computers:ti,ab OR tablet*:ti,ab OR iPad*:ti,ab OR laptop*:ti,ab OR iphone*:ti,ab OR iPhone*:ti,ab OR Android:ti,ab) |
| #3 | Population Group | ('adolescent'/de OR 'child'/de OR 'school child'/de OR adolescen*:ti,ab OR child:ti,ab OR children:ti,ab OR teen*:ti,ab OR boys:ti,ab OR boy:ti,ab OR girls:ti,ab OR girl:ti,ab OR youth:ti,ab OR youths:ti,ab) |
| #4 | Psychological Symptoms/Psychopathologies | ('somatoform disorder'/exp/mj OR 'eating disorder'/exp/mj OR 'obsessive compulsive disorder'/mj OR 'personality disorder'/exp/mj OR 'automutilation'/exp OR 'anorexia'/exp/mj OR 'anxiety'/exp/mj OR 'anxiety disorder'/exp/mj OR 'binge eating disorder'/exp/mj OR 'bipolar disorder'/exp/mj OR 'bulimia'/mj OR 'depression'/exp/mj OR 'dysthymia'/exp/mj OR 'fear'/exp/mj OR 'mania'/exp/mj OR 'panic'/mj OR 'phobia'/mj OR 'suicide'/exp/mj OR 'paranoid psychosis'/exp/mj OR 'psychosis'/exp/mj OR 'schizophrenia'/exp/mj OR 'attention deficit hyperactivity disorder'/exp/mj OR 'problem behavior'/exp/mj OR 'impulse control disorder'/exp/mj OR 'intermittent explosive disorder'/exp/mj OR 'aggression'/exp/mj OR 'antisocial personality disorder'/exp/mj OR 'conduct disorder'/exp/mj OR 'impulsiveness'/exp/mj OR 'substance abuse'/exp/mj OR 'drug dependence'/exp/mj OR 'communication disorder'/exp/mj OR 'developmental disorder'/exp/mj OR 'intellectual impairment'/exp/mj OR 'language disability'/exp/mj OR 'developmental language disorder'/exp/mj OR 'learning disorder'/exp/mj OR 'motor dysfunction'/exp/mj OR 'speech sound disorder'/exp/mj OR 'speech disorder'/exp/mj OR 'fluency disorder'/exp/mj OR 'stuttering'/exp/mj OR 'autism'/exp/mj OR 'Asperger syndrome'/exp/mj OR 'mental disease'/mj OR 'tic'/exp/mj OR 'Gilles de la Tourette syndrome'/exp/mj OR 'posttraumatic stress disorder'/exp/mj OR 'distress syndrome'/exp/mj OR 'gender dysphoria'/exp/mj OR 'psychosexual disorder'/exp/mj OR 'anorexia nervosa'/exp/mj OR somatoform:ti,ab OR 'eating disorder*':ti,ab OR 'eating patho*':ti,ab OR 'obsessive compulsive disorder*':ti,ab OR 'personality disorder*':ti,ab OR 'self harm*':ti,ab OR 'self-harm*':ti,ab OR 'self-injur*':ti,ab OR 'self injur*':ti,ab OR automutilat*:ti,ab OR 'self mutilate*':ti,ab OR 'self-multilat*':ti,ab OR 'self inflicted injur*':ti,ab OR 'sexual problem*':ti,ab OR 'gender dysphori*':ti,ab OR 'gender identity disorder*':ti,ab OR 'psychosexual disorder*':ti,ab OR anorex*:ti,ab OR anxiety:ti,ab OR anxieties:ti,ab OR anxious*:ti,ab OR 'binge eat*':ti,ab OR 'binge-eat*':ti,ab OR bipolar:ti,ab OR bulim*:ti,ab OR depressi*:ti,ab OR dysthymia*:ti,ab OR fear*:ti,ab OR hypomani*:ti,ab OR internaliz*:ti,ab OR mania*:ti,ab OR manic:ti,ab OR panic:ti,ab OR panics:ti,ab OR panicked:ti,ab OR phobi*:ti,ab OR suicid*:ti,ab OR trauma*:ti,ab OR 'thought disorder*':ti,ab OR paranoi*:ti,ab OR psychosis:ti,ab OR psychotic:ti,ab OR schizo*:ti,ab OR 'anger control':ti,ab OR 'anger management':ti,ab OR 'attention deficit*':ti,ab OR 'attention problem*':ti,ab OR 'behavior problem*':ti,ab OR 'behaviour problem*':ti,ab OR 'behavioral development*':ti,ab OR 'behavioural development*':ti,ab OR 'behavioral outcome*':ti,ab OR 'behavioural outcome*':ti,ab OR 'behavioral well-being':ti,ab OR 'behavioural well-being':ti,ab OR 'behavioral wellbeing':ti,ab OR 'behavioural wellbeing':ti,ab OR 'disruptive behavior*':ti,ab OR 'disruptive behaviour*':ti,ab OR 'intermittent explosive disorder*':ti,ab OR 'impulse control disorder*':ti,ab OR 'oppositional defiant disorder*':ti,ab OR aggress*:ti,ab OR antagonistic:ti,ab OR antisocial:ti,ab OR 'anti-social':ti,ab OR 'anti social':ti,ab OR 'conduct disorder*':ti,ab OR disinhibit*:ti,ab OR externaliz*:ti,ab OR hyperactiv*:ti,ab OR hyperkine*:ti,ab OR impulsiv*:ti,ab OR inattenti*:ti,ab OR irritab*:ti,ab OR sociopath*:ti,ab OR 'substance use*':ti,ab OR 'substance abuse*':ti,ab OR addict*:ti,ab OR 'drug dependenc*':ti,ab OR 'communication disorder*':ti,ab OR 'coordination disorder*':ti,ab OR 'developmental delay*':ti,ab OR 'developmental disorder*':ti,ab OR 'developmental disabilit*':ti,ab OR 'development disorder*':ti,ab OR 'fluency disorder*':ti,ab OR 'intellectual disab*':ti,ab OR 'intellectual impairment*':ti,ab OR 'language disorder*':ti,ab OR 'language development disorder*':ti,ab OR 'language developmental disorder*':ti,ab OR 'developmental language disorder*':ti,ab OR 'language disabilit*':ti,ab OR 'learning disorder*':ti,ab OR 'learning disabilit*':ti,ab OR 'motor disorder*':ti,ab OR 'movement disorder*':ti,ab OR 'motor dysfunction*':ti,ab OR 'speech sound disorder*':ti,ab OR 'speech disorder*':ti,ab OR 'articulation disorder*':ti,ab OR autis*:ti,ab OR asperger*:ti,ab OR 'neurodevelopmental disorder*':ti,ab OR 'neuro-developmental disorder*':ti,ab OR stutter*:ti,ab OR tic:ti,ab OR tics:ti,ab OR Tourette*:ti,ab OR 'posttraumatic stress disorder*':ti,ab OR 'post-traumatic stress disorder*':ti,ab OR 'post traumatic stress disorder*':ti,ab OR 'mental disorder*':ti,ab OR 'mental illness*':ti,ab OR 'psychological distress*':ti,ab OR 'distress syndrome*':ti,ab OR 'psychological impair*':ti,ab OR 'psychological symptom*':ti,ab OR psychopatho*:ti,ab) |
| #5 |  | #1 AND #2 AND #3 AND #4 |
| #6 | Limit applied: Language and Source | #5 AND [english]/lim AND ([embase]/lim OR [embase classic]/lim) |
|  | Limit applied: Exclude specific publication types | #6 NOT ([conference abstract]/lim OR [conference paper]/lim OR [conference review]/lim OR [data papers]/lim OR [editorial]/lim OR [erratum]/lim OR [letter]/lim OR [note]/lim OR [review]/lim OR [short survey]/lim OR [systematic review]/lim OR [meta analysis]/lim OR 'conference abstract'/exp OR 'conference paper'/exp OR 'data paper'/exp OR 'editorial'/exp OR 'letter'/exp OR 'erratum'/exp OR 'note'/exp OR 'short survey'/exp OR 'review'/exp OR 'systematic review'/exp OR corrigenda:ti,ab OR corrigendum:ti,ab OR erratum:ti,ab OR errata:ti,ab OR 'conference abstract*':ti,ab OR 'conference proceeding*':ti,ab OR symposium*:ti,ab OR letter:ti,ab OR editorial:ti,ab OR commentary:ti,ab OR 'meta analysis':ti,ab OR 'meta analyses':ti,ab OR metaanalysis:ti,ab OR metaanalyses:ti,ab OR metanalysis:ti,ab OR metanalyses:ti,ab OR 'meta-analysis':ti,ab OR 'meta-analyses':ti,ab OR 'systematic review*':ti,ab OR 'integrative review*':ti,ab OR 'case report*':ti,ab OR 'case series':ti,ab OR 'case study':ti,ab OR 'case studies':ti,ab OR 'case presentation*':ti,ab OR 'case stud*':ti,ab OR 'case histor*':ti,ab OR 'single case design*':ti,ab OR 'single-case*':ti,ab OR 'single-case analysis':ti,ab OR 'single cases':ti,ab OR 'case study'/exp OR 'single-case study'/exp) |

**Notes:** The limits for language (English) and sources (Embase and Embase Classic) were applied to the main search using the filters available in Embase. The keywords were searched in the title and abstract fields (i.e., :ti,ab), and the EMTREE controlled vocabulary terms were searched as /de or if the EMTREE term was exploded to automatically include all narrower terms this was indicated with /exp. Phrases were enclosed in single quotation marks to force the searching of the exact terms in order presented. A search strategy was used to exclude specific publication types as specified in the eligibility criteria. No other limits were applied to the searches.
