# Supplementary material for: Adherence to ecological momentary assessment studies in children and adolescents with psychopathology: A systematic review with meta-analysis
Source: NPP Digit Psychiatry Neurosci. 2026 Apr 27;4:9. doi: 10.1038/s44277-026-00058-z (PMC13121683; doi:10.1038/s44277-026-00058-z)
Supplement: Supplementary file 10 — Appendix C [file 44277_2026_58_MOESM10_ESM.docx]

**APPENDIX C**

Quality Assessment Checklist

*Adapted from Morren et al. [20]*

1. *Formulation of the Research Question*

Is the research question justified by previous literature presented in the introduction section? Are the hypotheses appropriate for the stated research question?

2 = Clear, directional, testable hypotheses grounded in previous literature

1 = More general hypotheses with some basis in previous literature

0 = No hypotheses or some hypotheses without clearly explained rationale

1. *Specification of Inclusion and Exclusion Criteria*

Is there a clear description of inclusion and exclusion criteria?

2 = Clearly stated inclusion and exclusion criteria that would provide basis for replication

1 = Inclusion and exclusion criteria stated but potentially difficult to replicate (due to missing or unclear information)

0 = Inclusion and exclusion criteria not presented

1. *Sampling*

Are recruitment aspects clearly described and appropriate for the research question? For example, an ideal recruitment description might include means of recruitment (flyers, listserv, website, etc.); target sample (community, clinical); location; and time at which recruitment occurred.

2 = Recruitment process clearly reported and detailed to allow for replication (most expected elements are covered)

1 = Elements of recruitment reported but not specific enough to allow for replication (some expected elements are covered)

0 = Recruitment aspects not sufficiently reported (none of the expected elements are covered)

1. *Description of Non-response*

Is overall adherence clearly described and is enough information provided to allow for calculation of compliance, enrollment, and drop-out?

2 = 3/3 aspects are clearly described

1 = 2/3 or 1/3 aspects are clearly described

0 = 0/3 aspects clearly described

1. *Design*

Does the study design allow for appropriate testing of the stated hypotheses?

2 = Study design has all necessary components to test the stated hypotheses

1 = Most elements of study design are appropriate to answer the research questions

0 = Study design is inappropriate to answer the research questions

1. *Sample Description*

Is the sample clearly described? Are you able to fully complete the data extraction items related to demographics (e.g., race/ethnicity, sex, age, etc.)? Take into account that reporting standards may differ based on country in which the sample is acquired.

2 = Sample is clearly described and consistent with the research question

1 = Sample description is missing some key elements, or sample is well described but not related to research question

0 = Sample description is insufficient or not present

1. *Reliability and Validity of Measures*

How well do the authors describe the validity and reliability of the measures they are using?

2 = Detailed effort to discuss the validity and reliability of measure in existing literature and good validity and reliability within their sample

1 = Some attempt to discuss validity and reliability, or validity and reliability of measures are well described but they are not sufficiently valid or reliable

0 = Insufficient or nonexistent discussion of validity and reliability

1. *Description of the Study Procedure*

Is the study comprehensive in describing all elements of study design?

2 = All elements are clearly described and detailed to allow for replication

1 = Some elements are clearly reported, or all are present but not detailed enough for replication

0 = Elements are not sufficiently reported

1. *Research Questions are Answered*

Is there congruence between the hypotheses and results for each of the questions in the results section?

2 = All of the questions are answered well

1 = Some of the questions are answered rather than all, or all are answered but not well

0 = One or fewer are answered, or a couple are answered but not well
