# Supplementary material for: Adherence to ecological momentary assessment studies in children and adolescents with psychopathology: A systematic review with meta-analysis
Source: NPP Digit Psychiatry Neurosci. 2026 Apr 27;4:9. doi: 10.1038/s44277-026-00058-z (PMC13121683; doi:10.1038/s44277-026-00058-z)
Supplement: Supplementary file 11 — Appendix D [file 44277_2026_58_MOESM11_ESM.pdf]

APPENDIX D

Funnel Plots by Outcome and Group

Enrollment

Full

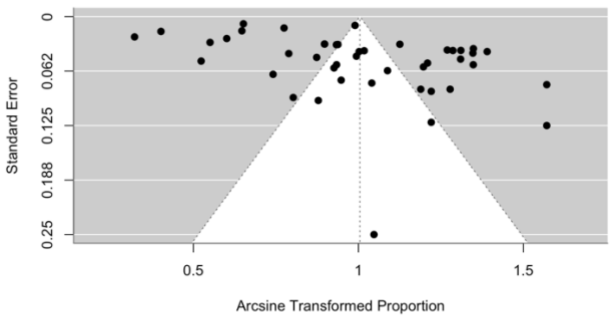

Community

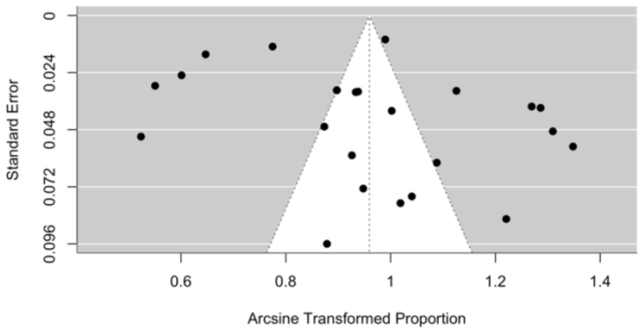

Healthy

NA

All Psychopathology

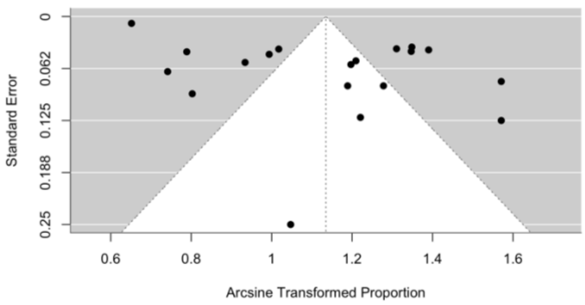

Internalizing

NA

Externalizing

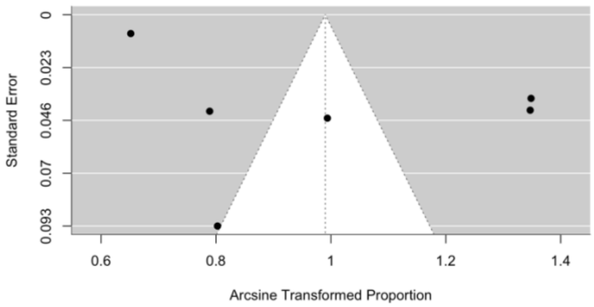

Neurodevelopmental

NA

Eating Pathology

NA

Thought Disorders

NA

Trauma Exposure/At Risk

NA

Somatic

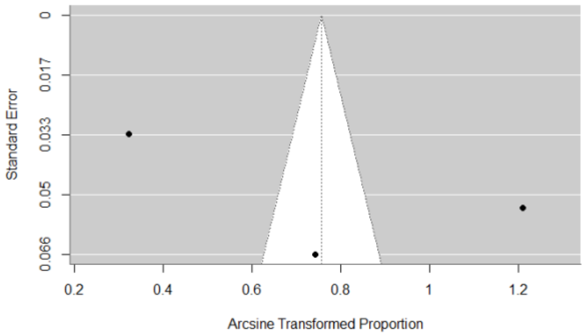

Standard Error
0
0.017
0.033
0.05
0.066
0.2
0.4
0.6
0.8
1
1.2
Arcsine Transformed Proportion

Dropout (No Participation)

Full

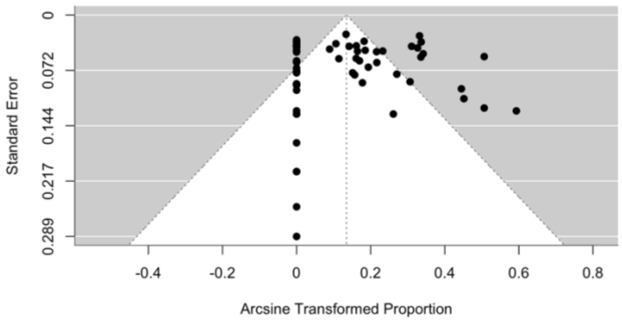

Community

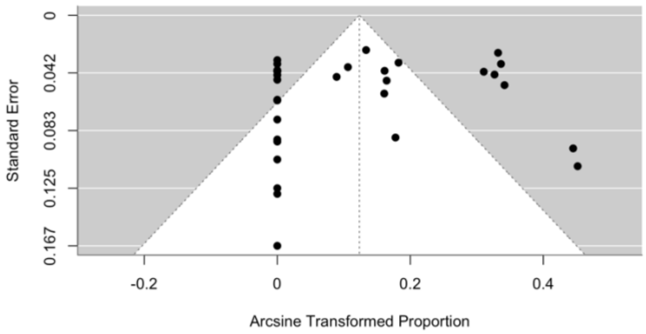

Healthy

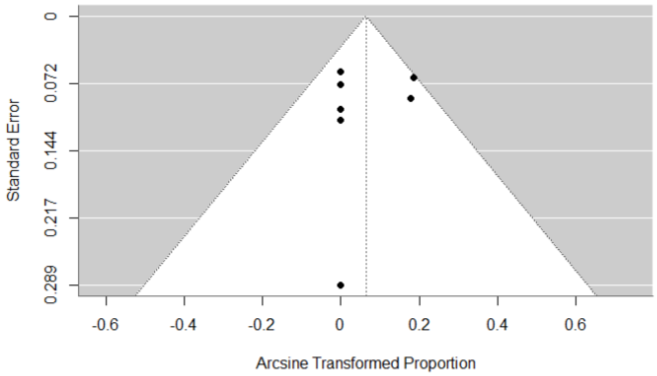

All Psychopathology

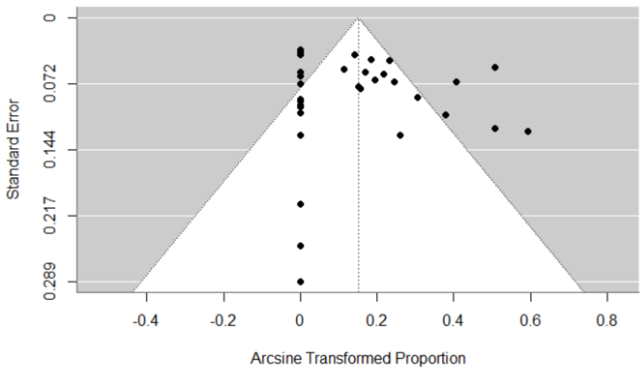

Internalizing

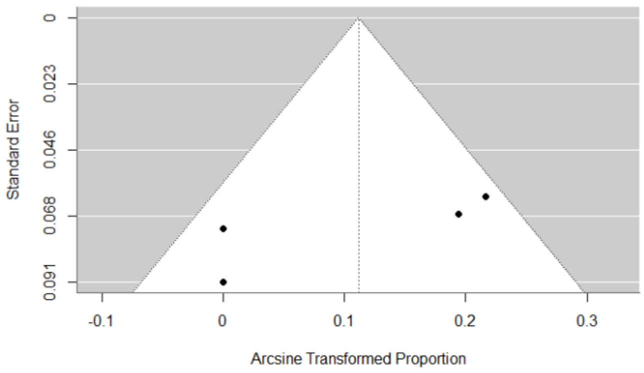

Externalizing

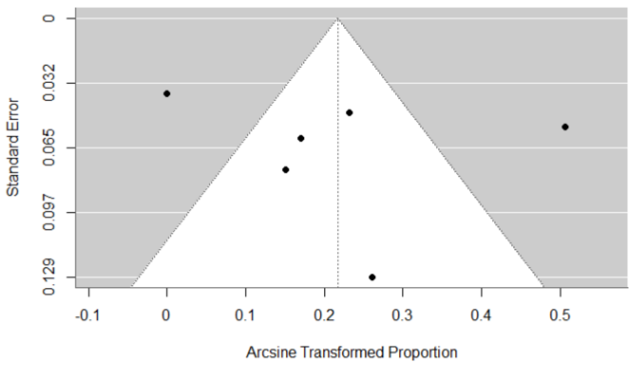

Neurodevelopmental

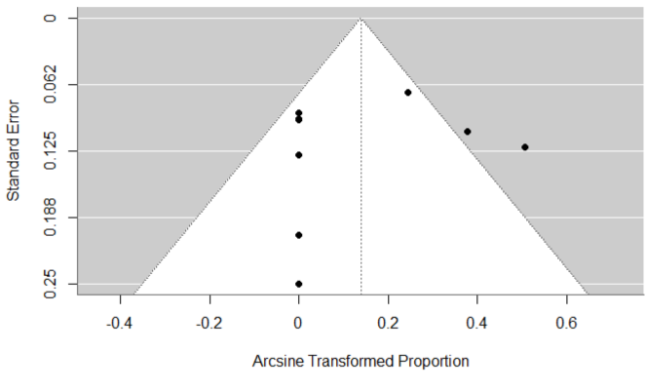

Eating Pathology

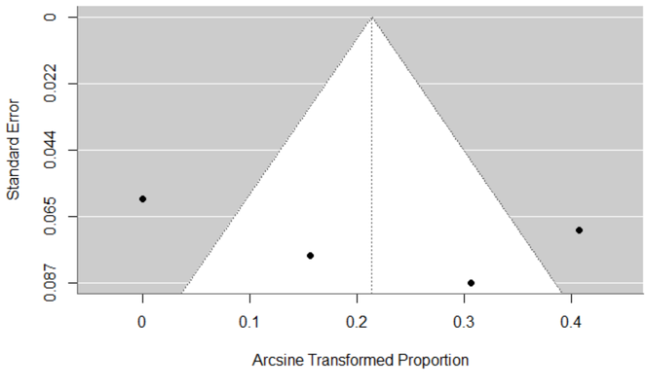

Thought Disorders

NA

Trauma Exposure/At Risk

NA

Somatic

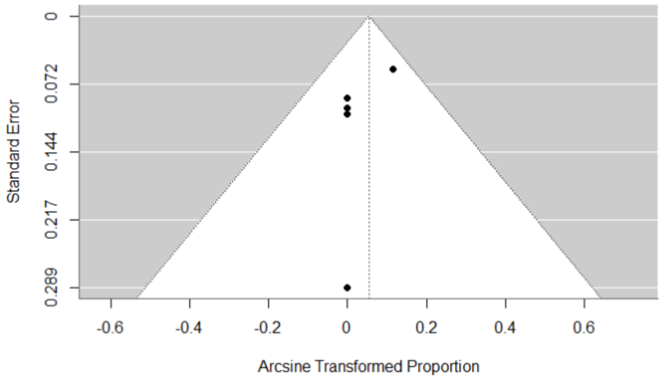

0
0.072
0.144
0.217
0.289
Standard Error
-0.6
-0.4
-0.2
0
0.2
0.4
0.6
Arcsine Transformed Proportion

Dropout (Some Participation)

Full

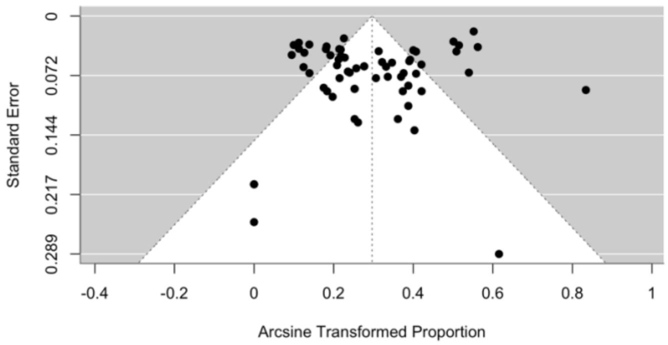

Community

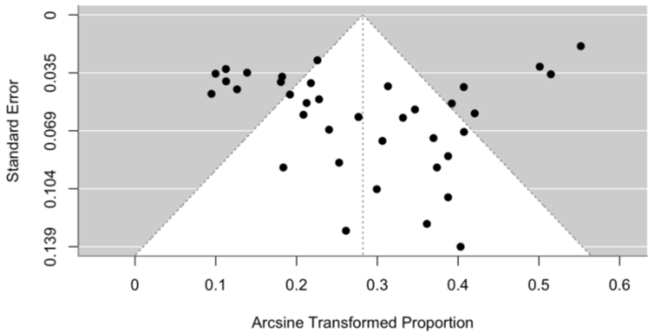

Healthy

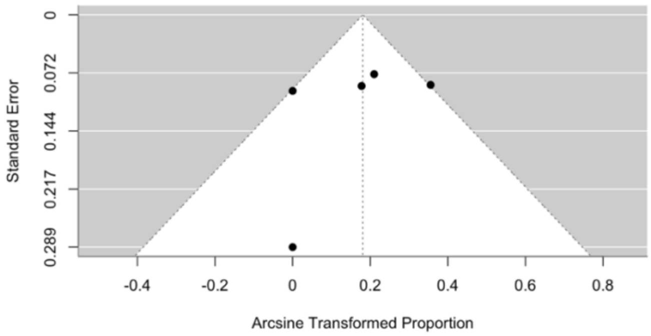

All Psychopathology

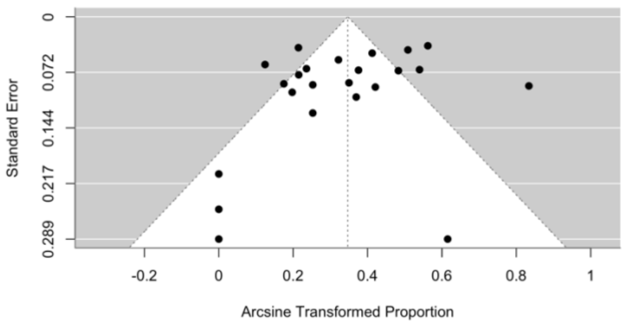

Internalizing

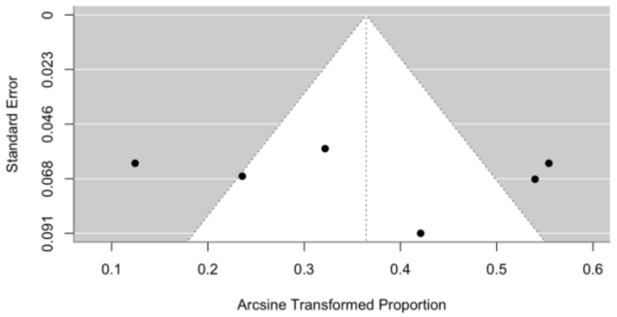

Externalizing

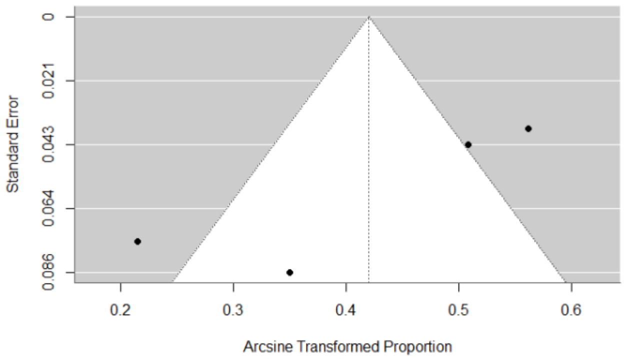

Neurodevelopmental

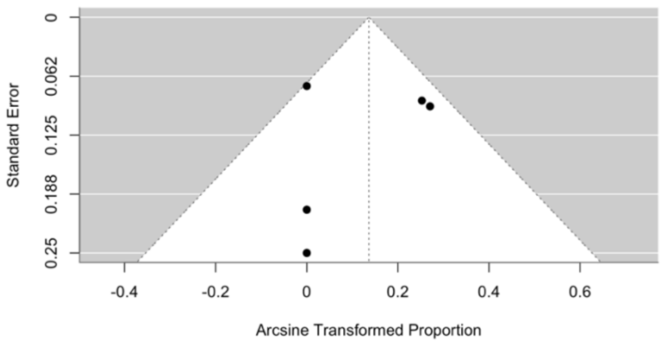

Eating Pathology

NA

Thought Disorders

NA

Trauma Exposure/At Risk

NA

Somatic

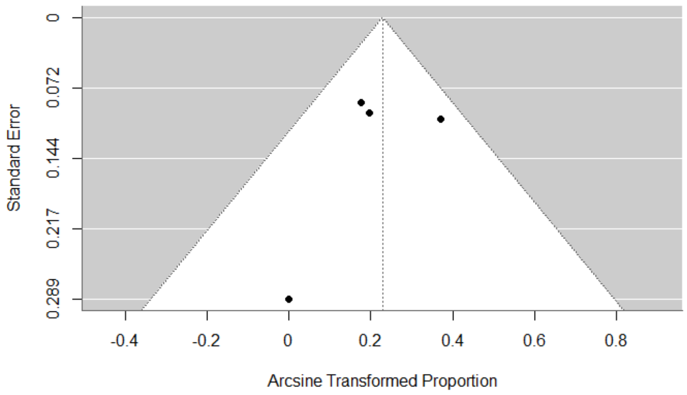

Compliance

Full

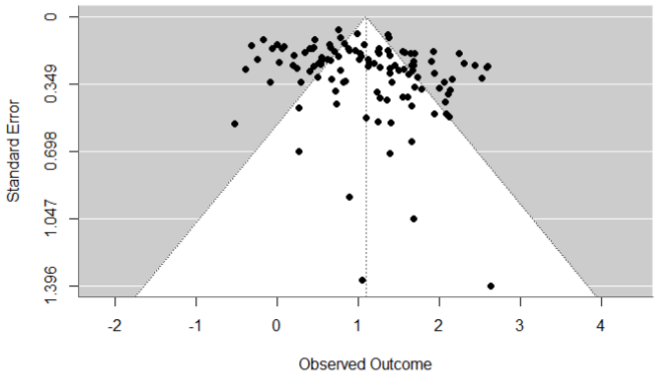

Community

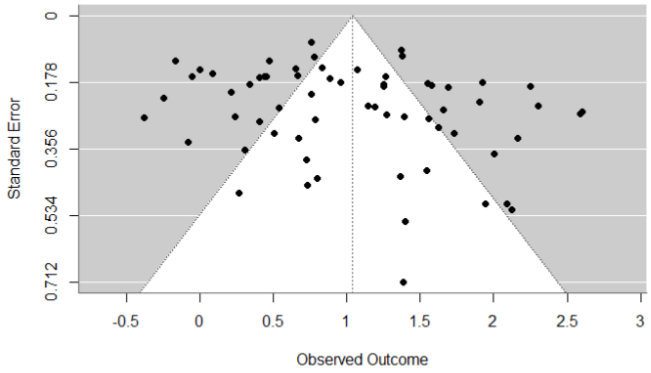

Healthy

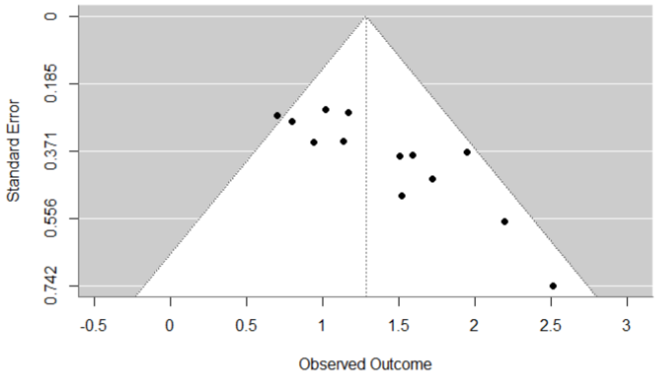

All Psychopathology

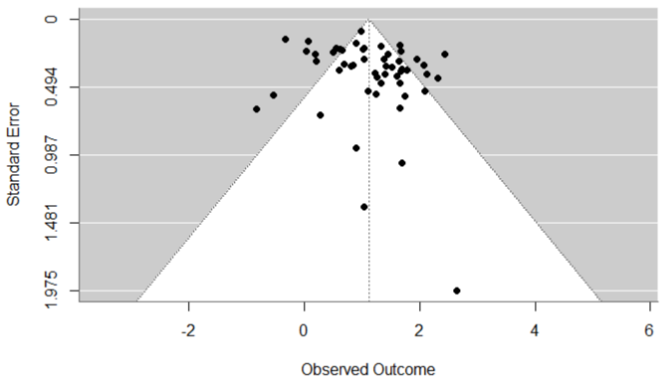

Internalizing

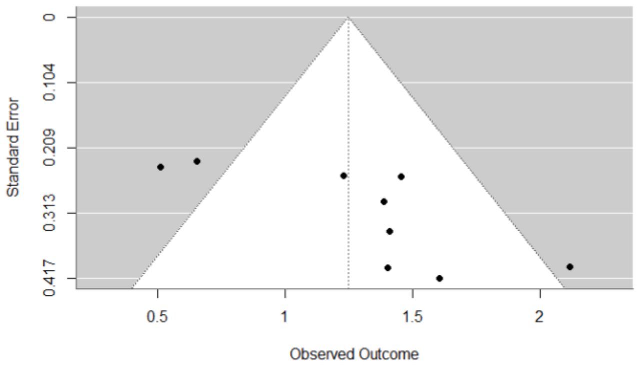

Externalizing

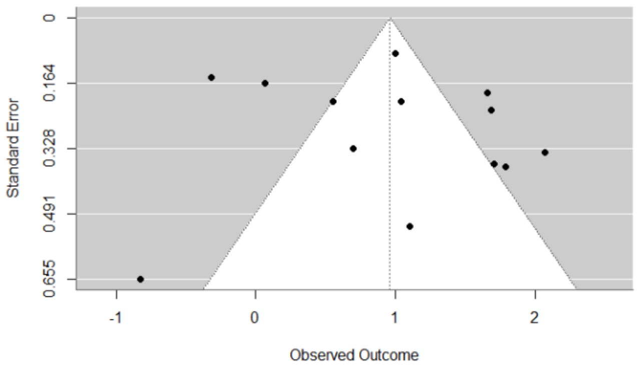

Neurodevelopmental

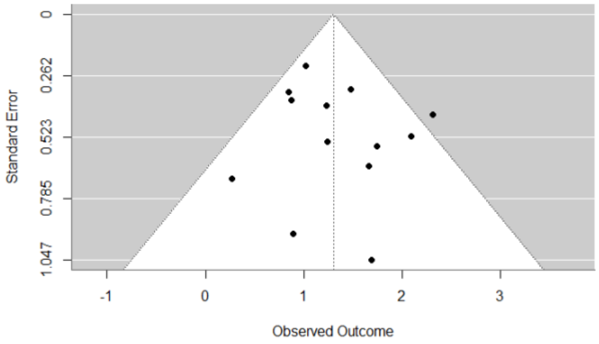

Eating Pathology

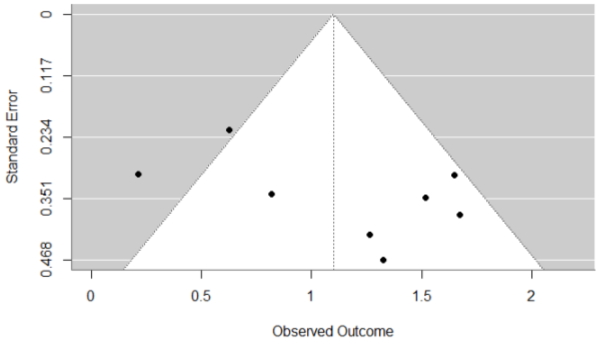

Thought Disorders

NA

Trauma Exposure/At Risk

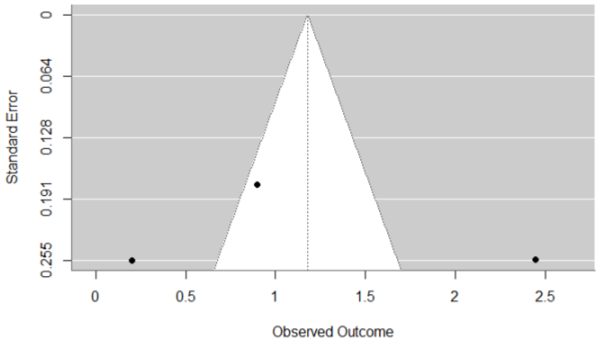

Somatic

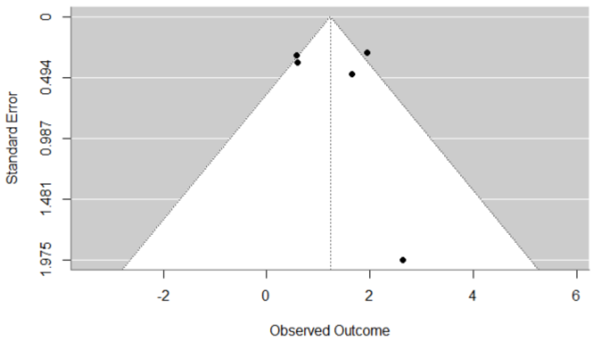

0
0.494
0.987
1.481
1.975
Standard Error
-2
0
2
4
6
Observed Outcome
